# Bond strength of debonded orthodontic brackets after different reconditioning and priming protocols: An in vitro study

**DOI:** 10.1371/journal.pone.0353265

**Published:** 2026-07-07

**Authors:** Nguyen Viet Anh, Nguyen Thi Thu Huong, Trinh Khanh Linh, Le Thi Lan Anh, Dao Ngoc Linh Chi, Nguyen Thi Thu Huong, Nguyen Thi Trang

**Affiliations:** 1 School of Dentistry, Hanoi Medical University, Hanoi, Vietnam; 2 Department of Prosthodontics, School of Dentistry, Hanoi Medical University, Hanoi, Vietnam; 3 Faculty of Dentistry, Phenikaa University, Hanoi, Vietnam; Danube Private University, AUSTRIA

## Abstract

**Introduction:**

Although various bracket recycling methods have been studied, the combined influence of bracket reconditioning methods and primer protocols on the bonding performance of debonded orthodontic brackets remains unclear. This study aimed to evaluate the effects of three reconditioning methods and three primer protocols on the bond strength of rebonded orthodontic brackets to enamel.

**Materials and methods:**

One hundred stainless steel brackets were bonded to extracted premolars and divided into 10 groups (n = 10). Ninety brackets were debonded, reconditioned using OneGloss bur, flaming, or sandblasting, and rebonded with Mani Bond, Denu Bond, or no primer. Ten new brackets served as controls. Bracket bases were examined using scanning electron microscopy (SEM). Bond strength, adhesive remnant index (ARI), and bonding reliability were evaluated using SBS testing, ARI scoring, and Weibull analysis.

**Results:**

Bond strength was significantly influenced by the interaction between reconditioning method and primer protocol (p < 0.001). Sandblasting combined with Mani Bond produced the highest bond strength (9.33 ± 3.02 MPa), whereas OneGloss and sandblasting combined with Denu Bond achieved bond strengths comparable to new brackets and higher reliability, reflected by greater Weibull moduli (4.74 and 4.72). SEM demonstrated more effective mesh cleaning after sandblasting than after OneGloss bur treatment or flaming. ARI distributions differed significantly among groups (p = 0.01), with failures occurring predominantly at the bracket–adhesive interface.

**Conclusions:**

The bonding performance of rebonded orthodontic brackets is influenced by the interaction between reconditioning and priming protocols. Appropriate combinations of these procedures may improve the rebonding outcome.

## Introduction

Orthodontic treatment corrects malocclusions through the use of various appliances, most commonly the preadjusted edgewise system, in which brackets are bonded from the maxillary to mandibular second premolars on either the labial or lingual surfaces [1,2]. These brackets act as the interface for delivering corrective forces, and their bonding strength is essential for maintaining appliance stability throughout treatment. Failures in bracket adhesion can prolong therapy and increase the clinical workload, as well as treatment cost [3,4], making optimal bonding protocols a key concern.

While some bracket failures are unavoidable and can be as high as 23.4% [4] especially in the posterior region or in the deep bite cases [3,5], brackets can also be detached intentionally from the tooth surface by orthodontists during treatment to correct undesirable tooth position or attain orthodontic purposes, to help the tooth move as planned [6]. Accurately positioning brackets on malaligned teeth presents a considerable challenge in orthodontic practice. Consequently, certain brackets may initially be placed incorrectly and require repositioning to achieve their ideal alignment, even when other brackets are already properly positioned.

Although there are some concerns with the recycling bracket, such as hygiene, time-consuming, distortion of the bracket slot, and alteration of the bracket mesh base [7–9], reusing debonded brackets can reduce treatment costs by up to 90% [10] and minimise environmental waste.

A variety of bracket recycling techniques have been described in the literature, including mechanical approaches (e.g., high- and low-speed handpieces, green stone burs) [11–13], sandblasting [14], chemical treatments [15,16], thermal methods [10], ultrasonic cleansing [10], laser therapy [17], and combinations of these strategies. Adhesive removal using burs and handpieces is convenient and does not require additional equipment; however, they are less effective in removing adhesive remnants and have been associated with reduced bond strength compared with sandblasting in some studies [18,19]. By contrast, sandblasting has been shown in multiple studies to efficiently remove residual adhesive in a short time, restore bond strength to optimal levels, and achieve this at relatively low equipment cost [10,18,20]. Recent in-vitro work comparing enamel reconditioning methods supports sandblasting as an effective approach to restore bond strength in rebonding scenarios [21].

The attachment of brackets to enamel relies not only on the mechanical retention afforded by the bracket mesh but also on the bonding system, which plays a crucial role in achieving durable bonding. Comparative in-vitro and in-vivo evaluations of self-etch and conventional primers show variable effects on bracket bond strength and should be considered when interpreting primer interactions [22]. While several studies have compared the bond strength of recycled brackets using different adhesives [23,24] and others have evaluated recycled brackets with or without primer [18], the specific influence of different primer systems on the bond strength of recycled brackets remains unexplored.

In evaluating orthodontic bonding performance, several key parameters are commonly used. Shear bond strength (SBS) reflects the force required to detach a bracket from the tooth surface and is widely used to assess the adequacy of bonding for clinical function. The adhesive remnant index (ARI) provides information on the site of bond failure by indicating the amount of adhesive remaining on the tooth after debonding, thereby offering insight into the bonding mechanism and potential risk of enamel damage [12]. In addition, bonding reliability can be assessed using Weibull analysis, which evaluates the consistency and predictability of bond strength across specimens [25]. Together, these parameters provide a comprehensive assessment of both the strength and durability of orthodontic bonding systems.

Given the growing focus on sustainability in orthodontics, bracket recycling represents an important strategy to minimize environmental impact [26,27]. Accordingly, evaluating bond strength under diverse reconditioning and primer protocols is necessary to maintain consistent clinical performance. Although several studies have evaluated bracket recycling methods individually, the combined influence of reconditioning techniques and primer systems on bond strength has not been explored. Therefore, this study aimed to determine whether different bracket reconditioning methods and primer protocols could restore the bonding performance of debonded orthodontic brackets, using new brackets as a reference control. The null hypothesis was that neither bracket reconditioning method nor primer protocol, nor their interaction, would significantly affect the bond strength of rebonded orthodontic brackets.

## Materials and methods

### Study design

Written informed consent was obtained from all patients who provided teeth for this study. Ethical approval for this study was obtained from the Phenikaa University Ethics Committee (Approval No. PUS2025-D6). The recruitment period lasted from 01/07/2025 to 15/09/2025. The required sample size was calculated using Statistical power analysis software (G*Power 3.1; Heinrich Heine University, Düsseldorf, Germany) (F tests, ANOVA: Fixed effects, omnibus, one-way) [28], assuming a medium effect size of 0.45, an alpha error of 0.05, and a statistical power of 80%. Accordingly, 10 teeth were allocated to each group.

### Sample preparation

Freshly extracted first premolars removed for orthodontic purposes were used. Teeth were cleaned under running water with a brush to remove debris, disinfected in 0.1% thymol solution for 48 hours, and then stored in distilled water to maintain hydration until testing. Each tooth was examined under dental illumination, and only those without cracks, fractures, occlusal caries, dentin dysplasia, or previous restorations were included. Teeth were embedded in self-curing acrylic resin (Pattern Resin LS; GC, Tokyo, Japan) at the cementoenamel junction to secure them to the SBS testing machine. Specimens were labelled by engraving the tooth number on the resin surface with a diamond bur for identification.

A total of 100 new stainless steel brackets (SmartLine; Medico, Seoul, South Korea) were used. The bracket base surface area, calculated with dental designing software (Medit Link; Medit, Seoul, South Korea), was 10.4 mm². The experiment included two variables: bracket cleansing method (three levels: OneGloss bur, flaming, sandblasting) and primer application (three levels: Mani Bond, Denu Bond, no primer). In addition, a control group consisting of new brackets bonded without primer application was included, resulting in a total of 10 groups with 10 brackets per group, as detailed in Table 1.

**Table 1:** Experimental design and bonding protocols for each study group.

Group	Initial bonding (tooth surface)	Reconditioning method	Rebonding agent (bracket base)
1	Denu Bond	OneGloss	Mani Bond
2		OneGloss	Denu Bond
3		OneGloss	None
4		Flaming	Mani Bond
5		Flaming	Denu Bond
6		Flaming	None
7		Sandblasting	Mani Bond
8		Sandblasting	Denu Bond
9		Sandblasting	None
10		Not applicable (New bracket)	None

Denu Bond was applied to the tooth surface for all groups during both bonding stages. The experimental variable was the primer applied to the bracket base during rebonding. Group 10 consisted of new brackets and served as the control group.

### Bonding and debonding procedure

Labial surfaces of the teeth were cleaned with fluoride-free pumice, etched with 37% phosphoric acid etchant (Total Etch; Ivoclar, Amherst, NY, USA) for 15 s, rinsed for 10 s, and gently air-dried for 10 s to achieve a frosty appearance. A bonding agent (Denu Bond; HDI, Seoul, South Korea) was applied for 20 s, air-dried for 5 s, and light-cured for 10 s using an LED curing unit (LED.F; Woodpecker, Guilin, China). Orthodontic adhesive (Transbond XT Light Cure Adhesive; 3M Unitek, Monrovia, CA, USA) was placed on the bracket base, and the bracket was bonded to the center of the tooth crown’s labial surface using bracket pliers under a standardised force of 5 N. Excess adhesive was removed, and the resin was light-cured for 40 s (10 s from each side) using an LED curing unit with an irradiance of 1000 mW/cm². During curing, the light guide tip was positioned as close as possible to the bracket surface (approximately 1 mm). The selected curing protocol was based on previous evidence demonstrating satisfactory orthodontic bonding performance with LED light-curing units operated at comparable irradiance levels and exposure times [29]. Bracket-bonded teeth were stored in distilled water at 37 °C for 24 h for water sorption and complete post-curing of adhesive resin. Subsequently, the specimens underwent thermocycling using a thermocycler (SD Mechatronik Thermocycler; Huber, Feldkirchen-Westerham, Germany) for 1,500 cycles between 5 °C and 55 °C, which has been reported to simulate approximately 7 weeks of short-term intraoral aging [30]. Each cycle consisted of 30 s immersion at each temperature with a 5 s transfer time between baths.

After bonding, 90 brackets were debonded with bracket removal pliers [12] and randomly assigned to Group 1–9. The remaining 10 brackets were left bonded as Group 10 and stored in distilled water at 37 °C until SBS testing.

### Bracket reconditioning and rebonding procedure

Brackets were reconditioned by three methods: OneGloss bur, flaming, and sandblasting.

- OneGloss group: Resin remnants on the bracket base were removed with a finishing and polishing bur (OneGloss; Shofu, Kyoto, Japan) in a low-speed handpiece at 20,000 rpm until no adhesive was visible macroscopically.
- Flaming group: Brackets were heated over a Bunsen burner (Paul Marienfeld, Lauda-Königshofen, Germany) for 5 s until the base turned red hot, as recommended in previous studies [10,18], then quenched in room-temperature water and air-dried.
- Sandblasting group: Brackets were first cleaned with a OneGloss bur to remove gross adhesive, then air-abraded with 50 µm aluminium oxide particles using a dental air polisher (M&Y Dental Air Polisher; M&Y Dental, Foshan, China). Sandblasting was applied at a 90° angle to the base, 1 cm distance, 0.3 MPa pressure, for 10 s [8]. Comparable studies have evaluated sandblasting combined with acid etching for rebonding and provide methodological precedent for particle size and pressure selection [31].

During the initial bonding procedure, all brackets were new; therefore, no primer was applied to the bracket base, and Denu Bond was applied only to the tooth surface for all specimens to standardize bonding conditions. Following debonding and bracket reconditioning, the experimental primer variable was introduced during rebonding. According to group allocation, a universal primer (Mani Bond; Mani, Tochigi, Japan), a fifth-generation primer (Denu Bond; HDI, Seoul, South Korea), or no primer was applied to the bracket base. Denu Bond was consistently applied to the tooth surface in all groups.

A thin primer layer was applied to the bracket base, left for 10 s, air-dried with oil- and water-free spray for 5 s, and light-cured for 10 s with an LED curing unit at 1000 mW/cm². Before placement, residual adhesive on the tooth surface was removed with a OneGloss bur, and brackets were bonded following the same protocol described above. Bracket-bonded teeth were stored in distilled water at 37 °C for 24 h before being subjected to SBS testing. Details of the primers and adhesive employed in this study are presented in Table 2.

**Table 2:** Chemical compositions of primers and adhesive used in the study.

Mani Bond	Mani, Japan	10-methacryloyloxydecyl dihydrogen phosphate (10-MDP);4-Methacryloxyethyltrimellitate anhydride (4-Meta); urethane diol dimethacrylate (UDMA); hydroxyethyl methacrylate (HEMA); hexanediol dimethacrylate (HDDMA) and bisphenol A-glycidyl methacrylate (Bis-GMA); ethanol; water; fumed silicas; Camphorquinone; acylphosphine oxide pH: Mild self-etching; pH > 2 (typically 2.0–2.5) Viscosity: Not reported by manufacturer
Denu Bond	HDI, Seoul, South Korea	Dimethacrylate resins; hydroxyethyl methacrylate HEMA; (1-methylethylidene) bis[4,1-phenyleneoxy(2-hydroxy-3,1-propanediyl)] bismethacrylate; 2,2′-ethylenedioxydiethyl dimethacrylate; 4-(dimethylamino) phenethyl alcohol; ethanol; water; fillers; initiators pH: Not reported by manufacturer Viscosity: Not reported by manufacturer
Transbond XT adhesive	3M, USA	Silanated quartz; bisphenol A glycidyl dimethacrylate; bisphenol A ethoxylate dimethacrylate; silanated silica; diphenyliodonium hexafluorophosphate; triphenylantimony

Composition and physicochemical properties are based on manufacturer-provided information. 10-MDP = 10-methacryloyloxydecyl dihydrogen phosphate; 4-META = 4-methacryloyloxyethyl trimellitate anhydride; UDMA = urethane dimethacrylate; HEMA = hydroxyethyl methacrylate; HDDMA = hexanediol dimethacrylate; Bis-GMA = bisphenol A glycidyl methacrylate.

### Shear bond strength (SBS) testing

Before SBS testing, the specimens were subjected to thermocycling (SD Mechatronik Thermocycler; Huber, Feldkirchen-Westerham, Germany) for 1,500 cycles between 5 °C and 55 °C, with a dwell time of 30 s at each temperature and a transfer time of 5 s. SBS was tested using a universal tensile testing machine (HD-B604-S; Haida Equipment, Dongguan, China). Each specimen was fixed in the clamp, and a 0.6 mm stainless steel wire (40 cm) was used to apply a shear force at the bracket–tooth interface at a crosshead speed of 1 mm/min. The wire showed 0.2% elongation under 400 N. Debonding force was recorded in Newtons, and SBS calculated in MPa by dividing this force by the bonded surface area (10.4 mm²). The examiner was blinded to group allocation to reduce bias.

### Adhesive remnant index (ARI)

Following the shear bond strength test, tooth surfaces were examined under a stereomicroscope (SZX16; Olympus, Tokyo, Japan) at 30× magnification to assess the percentage of residual adhesive. Group allocation was concealed from the examiner to prevent bias. Adhesive remnants on enamel were then scored according to previously described criteria [12]:

Score 0: No adhesive remains on the tooth surface.

Score 1: Less than 50% of the adhesive remains on the tooth surface.

Score 2: More than 50% of the adhesive remains on the tooth surface.

Score 3: All or nearly all adhesive remains on the tooth surface.

### SEM image analysis

Following bracket base cleansing, two brackets from each group were randomly selected for topographic evaluation with a scanning electron microscope (S-4800; Hitachi High-Technologies Corporation, Tokyo, Japan) at 20 kV and a 17.4-mm working distance. Before imaging, samples were coated with a conductive carbon layer by thermal evaporation and examined at ×150, ×300, and ×600 magnifications.

### Statistical analysis

Normality and variance homogeneity were assessed with the Shapiro–Wilk and Levene’s tests. A two-way factorial ANOVA with HC3 robust covariance estimation was used to assess the effects of bracket reconditioning method, primer protocol, and their interaction on SBS. Differences among the 10 experimental groups were additionally evaluated using one-way Welch ANOVA followed by Games–Howell post hoc comparisons. The chi-square test was applied to compare ARI score distributions among groups. Weibull analysis was used to evaluate bond strength reliability and variability, with the Weibull modulus (*m*), representing the consistency and predictability of bond strength, and the characteristic strength (σ₀) estimated by rank regression on the linearised distribution [25]. Higher m values indicate lower variability and greater bonding reliability. Differences in *m* (95% CI) were assessed with the pairwise confidence interval overlap method. Statistical significance was set at p < 0.05. Analyses were conducted in Python (v3.12.8; https://www.python.org)

## Results

### Shear bond strength (SBS)

The mean ± SD values of SBS for all experimental groups are summarised in Table 3.

**Table 3:** Shear bond strength (MPa) of experimental groups.

Group	Mean ± SD	Min	Max	95% CI	
**Lower Bound**	**Upper Bound**
1	3.13 ± 0.83	1.92	4.81	2.53	3.72
2	4.09 ± 1.00	2.88	5.77	3.38	4.80
3	2.35 ± 0.77	1.44	3.85	1.80	2.90
4	4.18 ± 1.26	2.88	6.25	3.28	5.09
5	3.75 ± 1.65	1.92	6.25	2.57	4.93
6	2.69 ± 1.25	0.96	3.85	1.80	3.59
7	9.33 ± 3.02	5.77	13.46	7.17	11.49
8	4.52 ± 1.02	3.37	6.73	3.79	5.25
9	2.35 ± 1.19	0.96	4.33	1.50	3.20
10	5.32 ± 1.74	3.65	8.65	4.08	6.56

Values are presented as mean ± standard deviation (SD), minimum, maximum, and 95% confidence interval (CI). Each group contained 10 specimens (n = 10). Groups 1–3: OneGloss bur reconditioning with Mani Bond, Denu Bond, or no primer; Groups 4–6: Flaming with Mani Bond, Denu Bond, or no primer; Groups 7–9: Sandblasting with Mani Bond, Denu Bond, or no primer; Group 10: New brackets without primer (control group).

Levene’s test indicated a violation of homogeneity of variance (W = 4.387, p < 0.01). Therefore, a two-way factorial ANOVA with heteroscedasticity-robust (HC3) was conducted to examine the effects of adhesive removal method and primer protocol on shear bond strength. The main effect of the adhesive removal method was not statistically significant (F = 0.84, df = 2, p = 0.43), indicating that bond strength did not differ significantly among the adhesive removal methods. In contrast, the main effect of primer was statistically significant (F = 3.30, df = 2, p = 0.04), suggesting that bond strength varied according to the primer protocol used. Furthermore, a significant interaction was found between adhesive removal method and primer (F = 7.90, df = 4, p < 0.001), indicating that the effect of primer on bond strength depended on the adhesive removal method employed (Table 4).

**Table 4:** Robust two-way factorial ANOVA results for shear bond strength (SBS).

Source	Type III Sum of Squares	df	F	p-value
Reconditioning method	3.69	2.00	0.84	0.43
Primer	14.46	2.00	3.30	0.04
Reconditioning method*Primer	69.14	4.00	7.90	<0.001

Results of the robust two-way factorial ANOVA evaluating the effects of bracket reconditioning method, primer protocol, and their interaction on shear bond strength (SBS). Degrees of freedom (df), F-statistics, and p-values obtained using heteroscedasticity-robust (HC3) covariance estimation are presented. Statistical significance was set at p < 0.05.

**Fig 1:**
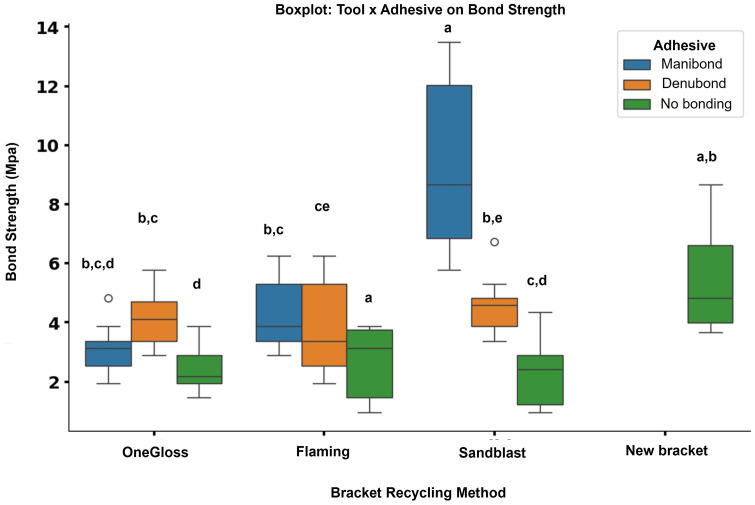
Shear bond strength of the study groups (MPa). Bars sharing the same letter indicate no significant difference in SBS values (p > 0.05; one-way Welch ANOVA with Games–Howell post hoc test).

At the group level, one-way Welch’s ANOVA confirmed significant differences in SBS across the 10 experimental groups (p < 0.01). Post-hoc analysis revealed that Group 7 (sandblasting + Mani Bond) achieved the highest SBS (9.33 ± 3.02 MPa), significantly greater than all other groups (p < 0.05). Groups 1, 2, 4, 5, and 8 exhibited bond strength comparable to the control group of new brackets (Group 10) (p > 0.05) (Fig 1).

### Weibull analysis

Weibull analysis was conducted to assess the variability and reliability of bond strength across 10 groups (Table 5).

**Table 5:** Weibull analysis results.

Group	Weibull modulus (*m*) (95% CI)	Characteristic strength (σ₀) (95% CI)
1	4.18 (3.28–9.47)	3.42 (2.88–3.94)
2	4.74 (3.8–8.38)	4.47 (3.81–5.06)
3	3.47 (2.73–6.63)	2.62 (2.13–3.1)
4	3.74 (3.24–9.21)	4.65 (3.74–5.45)
5	2.62 (2.16–4.5)	4.25 (3.15–5.27)
6	2.54 (1.69–9.12)	3.20 (2.23–3.62)
7	3.62 (3.0–6.14)	10.40 (8.44–12.13)
8	4.72 (3.75–11.88)	4.91 (4.23–5.6)
9	2.25 (1.7–4.23)	2.71 (1.86–3.41)
10	3.4 (2.92–9.35)	5.94 (4.65–7.06)

Values are presented as Weibull modulus (*m*) and characteristic strength (σ₀, MPa) with 95% confidence intervals (CI). Higher Weibull modulus values indicate greater bond reliability and lower variability in bond strength. There was no significant difference in the Weibull modulus among groups (p < 0.05).

The highest characteristic strength (σ₀) was observed in Group 7 (10.4 MPa), whereas the lowest values were recorded in Groups 3 (2.62 MPa) and 9 (2.71 MPa).

Although the differences were not statistically significant, the Weibull modulus (*m*), which reflects the reliability and consistency of bond strength, varied among the study groups. Groups 5, 6, and 9 exhibited the lowest Weibull moduli (m < 2.6), suggesting greater variability and less predictable bonding performance. In contrast, Groups 2 (OneGloss + Denu Bond) and 8 (Sandblasting + Denu Bond) showed higher Weibull moduli (m > 4.5), indicating more consistent bond strength and greater bonding reliability, with a lower probability of premature bracket failure.

### ARI

**Fig 2:**
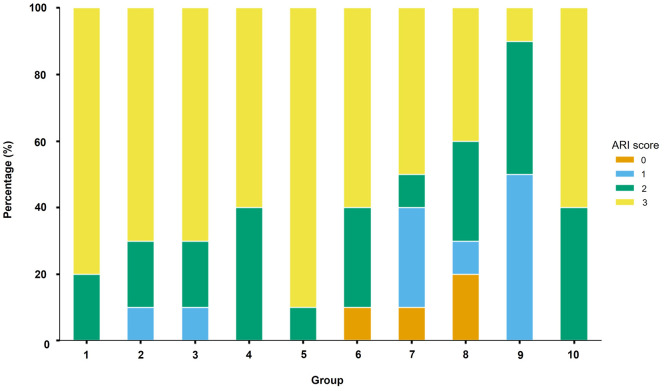
Distribution of Adhesive Remnant Index (ARI) scores among the experimental groups.

The distribution of the ARI score across the group was presented in Fig 2 and Table 6.

**Table 6:** Distribution of adhesive remnant index (ARI) scores among study groups.

Group	ARI score (%)				Total	p-value
**0**	**1**	**2**	**3**
1	0 (0%)	0 (0%)	2 (20%)	8 (75%)	10 (100%)	0.01
2	0 (0%)	1 (10%)	2 (20%)	7 (70%)	10 (100%)	
3	0 (0%)	1 (10%)	2 (20%)	7 (70%)	10 (100%)	
4	0 (0%)	0 (0%)	4 (40%)	6 (60%)	10 (100%)	
5	0 (0%)	0 (0%)	1(10%)	9 (90%)	10 (100%)	
6	1 (10%)	0 (0%)	3 (30%)	6 (60%)	10 (100%)	
7	1 (10%)	3 (30%)	1 (10%)	5 (50%)	10 (100%)	
8	2 (20%)	1 (10%)	3 (30%)	4 (40%)	10 (100%)	
9	0 (0%)	5 (50%)	4 (40%)	1 (10%)	10 (100%)	
10	0 (0%)	0(0%)	4 (40%)	6 (60%)	10 (100%)	

ARI = Adhesive Remnant Index. Score 0 = no adhesive remaining on the tooth surface; Score 1 = less than 50% adhesive remaining; Score 2 = more than 50% adhesive remaining; Score 3 = all or nearly all adhesive remaining on the tooth surface. Data are presented as the number (%) of specimens per group (n = 10). Differences in ARI score distribution among groups were analyzed using a Monte Carlo permutation test.

Because the chi-square test assumptions were violated (75% of expected cell counts < 5), exact methods were used. Differences in ARI score distributions among groups were assessed using a Monte Carlo permutation test. A significant association was observed between group and ARI score (p = 0.01). In most groups, ARI = 3 was the predominant score (ranging from 60–90% of samples). In contrast, groups 7–9 (sandblasting groups) showed lower proportions of ARI = 3 (10–50%), with ARI scores of 0–2 accounting for 50–90% of samples.

### SEM

**Fig 3:**
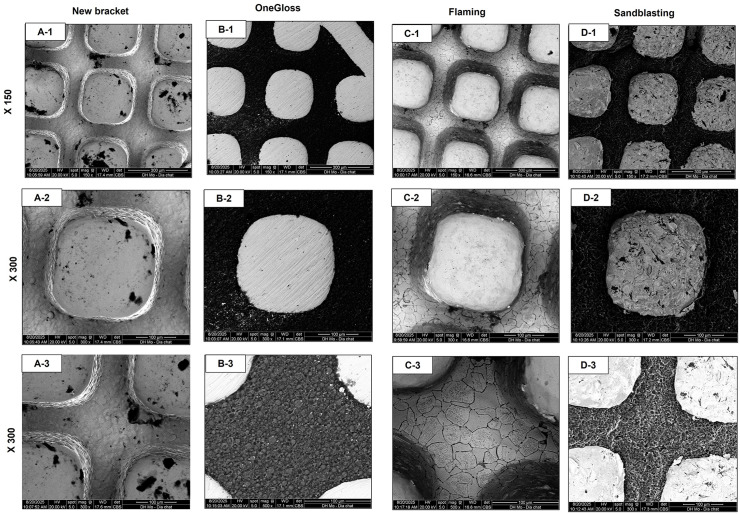
Scanning electron microscope (SEM) image analysis. SEM images of bracket bases: new brackets (A-1, A-2, A-3) and recycled brackets treated with OneGloss (B-1, B-2, B-3), flaming (C-1, C-2, C-3), and sandblasting (D-1, D-2, D-3). Images were captured at ×150 magnification (row 1), ×300 magnification (row 2) and ×600 magnification (row 3).

The SEM micrograph of the new bracket base revealed a regular mesh of square retentive units bordered by elevated ridges, forming undercuts and interspaces for micromechanical retention. The inner mesh surfaces appeared relatively smooth. Following reconditioning, all three methods preserved the overall mesh architecture, although differences in surface morphology were observed. After cleansing with OneGloss, the mesh pattern was less distinct due to resin remnants in the undercuts, while metallic surfaces appeared smoother with fine polishing striations. After flaming treatment, the original mesh was obscured by irregular residues, and the surfaces appeared rough and non-uniform with cracks, voids, and distortion of the mesh ridges. Following sandblasting, the mesh structure remained intact, but surfaces became rougher and irregular; undercuts appeared more pronounced, and adhesive remnants were largely removed (Fig 3).

## Discussion

This study aimed to evaluate the combined effects of bracket base reconditioning methods and primer protocols on the shear bond strength of orthodontic brackets. The findings showed that the primer protocol significantly affected bond strength, whereas the reconditioning method alone did not. However, a significant interaction between these factors indicated that the effect of the primer protocol depended on the reconditioning method employed. Among the combinations tested, sandblasting followed by application of Mani Bond produced the highest shear bond strength. Accordingly, the null hypothesis was partially rejected, as bond strength was significantly affected by the primer protocol and its interaction with the reconditioning method, but not by the reconditioning method independently.

When considered as an independent factor, the adhesive removal method did not significantly influence the bond strength of recycled brackets to the tooth surface. This finding contrasts with several previous studies comparing sandblasting and flaming, which reported superior shear bond strength following sandblasting treatment [18,32,33]. One possible explanation for this discrepancy is the difference in sandblasting protocols. In the present study, bracket bases were treated with 50-µm aluminum oxide particles applied from a distance of 10 mm for 10 seconds. In contrast, previous studies used either larger abrasive particles (90-µm aluminum oxide) [33] or different sandblasting parameters, such as a shorter application distance (5 mm) and longer treatment durations (20–30 seconds) [18,32]. SEM observations revealed distinct surface characteristics following the different adhesive removal methods. Sandblasting produced more complete adhesive removal and preserved a well-defined mesh structure, whereas OneGloss and flaming left residual resin within some undercut areas. In addition, sandblasting resulted in a rougher and more irregular metallic surface with slight erosion of the elevated ridges. However, despite these morphological differences, the reconditioning method alone did not significantly affect bond strength, suggesting that changes in bracket base topography were not sufficient by themselves to determine bonding performance. Instead, the interaction between bracket reconditioning and primer protocol appeared to play a more important role in influencing bond strength.

In the group of flame-treated brackets, the application of primer resulted in SBS levels comparable to those of new brackets without primer. A previous study reported similar findings, showing that flaming alone produced lower SBS than both sandblasting and new brackets when no primer was used [10]. However, when combined with primer, flaming achieved SBS values comparable to new brackets [34]. SEM analysis revealed that in the flaming group, the bracket base became rough and irregular, and evidence of surface distortion. Novianti et al. further reported that brackets subjected to flaming exhibited reduced resistance to erosion [35]. Therefore, although this method requires minimal equipment and can achieve SBS values comparable to those of new brackets, its adverse effects on bracket morphology and resistance to wear should not be overlooked, particularly in cases of repeated reuse.

Although no previous studies have specifically investigated OneGloss for bracket recycling, it is composed of aluminium oxide particles and is primarily designed as a polishing material. One rationale for choosing OneGloss over a metal bur is its ability to selectively remove residual composite without grinding the metal, thereby preserving the integrity of the bracket base. However, while it can effectively eliminate adhesive remnants, its polishing action tends to reduce surface roughness [36–38], which may in turn diminish mechanical retention. SEM observations in this study confirmed that OneGloss left residual resin within the undercut areas of the bracket base and produced a relatively smooth surface characterized by parallel polishing striations.

It should be noted that the measured SBS reflects the performance of the entire bracket–adhesive–enamel interface rather than the bracket base alone. Consequently, the adhesive–enamel bond also contributes to the observed bond strength values. Factors such as enamel morphology, acid-etching effectiveness, and resin infiltration may influence bonding performance. However, because enamel preparation, etching, adhesive application, and bonding procedures were standardized across all groups, differences in SBS are more likely related to the bracket reconditioning and priming protocols investigated in this study. This interpretation is supported by the ARI findings, which indicate that bond failure occurred at different locations within the bracket–adhesive–enamel interface depending on the experimental group. The predominance of ARI score 3 in most groups suggests that failure occurred mainly at the bracket–adhesive interface, whereas the greater proportion of ARI scores 0–2 in Groups 7–9 indicates increased involvement of the adhesive–enamel interface or mixed failure modes.

The results of the two-way analysis demonstrated a significant interaction between bracket reconditioning method and primer protocol, indicating that the effect of one factor depended on the other. Notably, the sandblasting–Mani Bond combination produced the highest SBS and favorable bonding reliability. However, interpretation of these findings should consider that SBS values are highly influenced by testing methodology, substrate characteristics, specimen preparation, and experimental conditions, resulting in substantial variability among studies [39,40]. Furthermore, the relationship between in vitro bond strength measurements and clinical performance is not straightforward [41]. Therefore, comparison with the new-bracket control group tested under identical conditions may provide a more meaningful benchmark than reliance on a universal SBS threshold alone. From this perspective, the sandblasting–Denu Bond combination achieved bond strengths comparable to those of new brackets, whereas the sandblasting–Mani Bond combination produced significantly higher SBS values. These findings suggest that both protocols were effective in improving the bonding performance of debonded brackets under the conditions of the present study, despite differences in their absolute SBS values. Previous studies reported that the use of primer significantly enhanced the shear bond strength of metal brackets compared to those bonded without primer application [18,42]. In addition, different universal and self-etch adhesives, owing to their distinct chemical compositions, have been shown to influence the bond strength of orthodontic brackets to tooth surfaces [43–45]. These universal primers contain functional acidic monomers (such as 10-MDP (10-methacryloyloxydecyl dihydrogen phosphate)) that enable chemical bonding with inorganic fillers and metal oxides present on restorative material [46,47]. This mechanism may explain the enhanced adhesion of metal brackets to the adhesive resin.

Weibull analysis indicated clear differences in both reliability and bond strength among the recycling protocols. OneGloss groups (1–3) achieved relatively high moduli (*m* ≈ 3.5–4.8), suggesting consistent bonding, but their characteristic strengths remained low (σ₀ = 2.62–4.47 MPa), limiting clinical effectiveness. Flaming groups (4–6) produced characteristic strength values comparable to the new bracket (up to 4.64 MPa when combined with primer), yet exhibited the lowest moduli (*m* = 2.5–3.6), indicating poor reliability. This reduced Weibull modulus may result from uneven adhesive remnants or bracket deformation caused by the burning process. In contrast, the sandblasting groups (Groups 7–9) exhibited variable bonding performance. Group 7 (sandblasting–Mani Bond) achieved the highest bond strength but only moderate reliability (*m* = 3.62), whereas Group 8 (sandblasting–Denu Bond) demonstrated bond strength comparable to that of new brackets and a higher Weibull modulus than Group 7, indicating greater bonding reliability. However, the difference in Weibull modulus between the two groups was not statistically significant.

Among the recycling protocols tested, sandblasting combined with a universal primer (Mani Bond) produced the highest bond strength, even exceeding that of new brackets. When paired with Denu Bond, sandblasting yielded bond strength comparable to new brackets but with greater consistency, as indicated by a higher Weibull modulus. Thus, sandblasting, when combined with an appropriate primer, can restore or even enhance bracket adhesion, offering a practical and sustainable option in clinical orthodontics. However, although higher bond strength is generally desirable to minimize bracket failure during treatment, excessively high SBS values may not always be clinically advantageous. Previous studies have suggested that bond strengths exceeding the level required to withstand orthodontic forces may increase the risk of enamel cracks, enamel fracture, or difficulty during bracket removal [48,49]. Therefore, an optimal bonding protocol should provide sufficient adhesion to withstand masticatory and orthodontic forces while allowing safe, controlled debonding at the end of treatment. Notably, although the sandblasting + Mani Bond group achieved the highest SBS in the present study (9.33 ± 3.02 MPa), this value remained below the threshold (14 MPa) previously associated with an increased risk of enamel damage during debonding [49]. Consequently, the enhanced bond strength observed with this protocol is unlikely to compromise enamel integrity while still providing reliable bracket retention. Nevertheless, the relationship between in vitro bond strength and clinical performance should be interpreted with caution, and further studies are required to determine the clinical relevance of these findings.

The present study has several strengths. First, both bracket reconditioning methods and primer protocols were evaluated within the same experimental design, allowing assessment of their individual effects as well as their interaction on the bonding performance of rebonded orthodontic brackets. Second, in addition to conventional bond strength testing, bonding reliability was assessed using Weibull analysis, providing complementary information on the consistency and predictability of bond performance that cannot be obtained from mean bond strength values alone. However, several limitations should be considered when interpreting the present findings. First, the SEM evaluation was performed on only two representative bracket bases per group and was intended to provide qualitative observations of surface morphology. No quantitative surface characterization, such as surface roughness analysis or energy-dispersive X-ray spectroscopy (EDS) [50], was performed. Therefore, the proposed mechanisms underlying the observed differences in bond strength should be interpreted with caution. Future studies incorporating larger SEM sample sizes and quantitative surface analyses are warranted. In addition, although the sandblasting group combined with Mani Bond produced the highest SBS values, the relatively small Weibull modulus (*m* = 3.62) indicates wide variability in bond strength, suggesting reduced reliability and predictability of the adhesive performance [25]. SEM images also revealed signs of wear and defects on the bracket surface. Previous research has shown that aluminium oxide particles possess irregular shapes with sharp edges, which can alter the bracket base upon impact [51]. Thus, the low Weibull modulus may be attributed to the influence of aluminium oxide particles on the surface morphology. Future studies comparing aluminium oxide with other particles of different shapes or with laser treatment would provide a more comprehensive view and stronger evidence regarding the most effective surface treatment method, especially for repeated bracket reuse.

## Conclusions

The reconditioning method alone did not independently determine bond strength outcomes; rather, the bonding performance of recycled orthodontic brackets was influenced by the interaction between reconditioning method and primer protocol. Among the protocols evaluated, sandblasting combined with Mani Bond produced the highest bond strength, whereas sandblasting or OneGloss reconditioning followed by Denu Bond application achieved bond strengths comparable to those of new brackets while maintaining favorable bonding reliability. These findings suggest that appropriate combinations of reconditioning and primer protocols can effectively improve the bonding performance of recycled brackets. Future studies should investigate residual surface elemental composition following different reconditioning procedures and evaluate alternative abrasive media or laser conditioning techniques to further optimize bracket recycling protocols.

## Supporting information

S1 File Supplementary files. (RAR)
