# Supplementary material for: Bond strength of debonded orthodontic brackets after different reconditioning and priming protocols: An in vitro study
Source: PLoS One. 2026 Jul 7;21(7):e0353265. doi: 10.1371/journal.pone.0353265 (PMC13340843; doi:10.1371/journal.pone.0353265)
Supplement: Attachment — Submitted filename: Reviewer_Report_PONE-D-25-59612_Bond_Strength.docx [file pone.0353265.s002.docx]

**Reviewer Report**

**Manuscript ID:** PONE-D-25-59612
**Title:** *Bond Strength of Recycled Orthodontic Brackets After Different Reconditioning and*

*Priming Protocols*

This manuscript presents an in vitro experimental evaluation of orthodontic bracket recycling techniques, examining how three reconditioning methods (OneGloss bur, flaming, sandblasting) and three primer systems influence shear bond strength, adhesive failure patterns, and bonding reliability. The study is well designed, clinically relevant, and contributes meaningful findings toward sustainable orthodontic practice. The study’s conclusions are supported by complete underlying data, tables, and well-presented figures that enhance transparency and allow clear visualization of results.

The manuscript is suitable for PLOS ONE and is generally well written, few clarifications and refinements would further strengthen scientific rigor and clarity.

**Recommendation:** Accept with Minor Revisions

**Comments for the Authors**

**Comment 1** – Suggest adding some definitions and importance of performance indicator parameters (i.e - SBS, ARI, bonding reliability etc), this could make the manuscript more coherent and user-friendly for broader audience.

**Comment 2** – Discussion of Potential Enamel Risk with High SBS Groups

Location: *Section 4, Page 20, Lines 337–340*
While mentioned briefly in the Discussion, please expand on the clinical implications regarding enamel crack risk during debonding, and the balance between strong adhesion and safe removal procedures.

**Comment 3** – Weibull Analysis Explanation

Location: *Section 3.2 – Weibull Analysis, Page 12–13, Lines 224–235*
The Weibull analysis is appropriate and informative. To improve clarity for readers, please add a brief explanation on how modulus *m* relates to clinical reliability

**Comment 4** – Minor Formatting Improvements

A light editorial pass is recommended to ensure consistency in:

- Figure numbering and caption style (Figures 1–3)
- Table formatting

These edits will improve readability and professional presentation.
