# Supplementary material for: Bond strength of debonded orthodontic brackets after different reconditioning and priming protocols: An in vitro study
Source: PLoS One. 2026 Jul 7;21(7):e0353265. doi: 10.1371/journal.pone.0353265 (PMC13340843; doi:10.1371/journal.pone.0353265)
Supplement: Attachment — Submitted filename: Response to Reviewers.docx [file pone.0353265.s004.docx]

**Manuscript ID. PONE-D-25-59612**

**Response to Reviewers**

Dear Editorial Board

*PLOS ONE*

Thank you for giving us the opportunity to submit a revised draft of the manuscript “Bond Strength of Recycled Orthodontic Brackets After Different Reconditioning and Priming Protocols” for publication in the *PLOS ONE*. We appreciate the time and effort you and the reviewers dedicated to providing feedback on our manuscript and are grateful for the insightful comments and valuable improvements to our paper.

We have incorporated most of the suggestions made by the reviewers. Those changes are highlighted within the manuscript in red. Please see below, in blue, for a point-by-point response to the reviewers’ comments and concerns. All page numbers refer to the revised manuscript file with tracked changes.

**Reviewer #1: Comments for the Authors**

1. Comment 1 – Suggest adding some definitions and importance of performance indicator parameters (i.e - SBS, ARI, bonding reliability etc), this could make the manuscript more coherent and user-friendly for broader audience.
**Author response:** We thank the reviewer for this helpful suggestion. To improve clarity and accessibility for a broader readership, we have added brief definitions and clinical relevance of key performance indicators, including shear bond strength (SBS), adhesive remnant index (ARI), and bonding reliability (Weibull analysis), in the Introduction section. These additions aim to enhance the coherence of the manuscript and facilitate interpretation of the results.

Please refer to the corresponding revisions in the manuscript (page 4, lines 101-110)
2. Comment 2 – Discussion of Potential Enamel Risk with High SBS Groups
Location: Section 4, Page 20, Lines 337–340
While mentioned briefly in the Discussion, please expand on the clinical implications regarding enamel crack risk during debonding, and the balance between strong adhesion and safe removal procedures.
**Author response:** We thank the reviewer for this valuable suggestion. We agree that the clinical implications of excessively high bond strength warrant further discussion. Accordingly, we have expanded the Discussion section to address the balance between achieving sufficient bond strength for clinical performance and avoiding excessively high bond strength that may increase the risk of enamel damage during bracket removal. We now discuss the potential for enamel cracks associated with high debonding forces, the importance of selecting bonding protocols that provide adequate but not excessive adhesion.

Please refer to the revised text that has been added to the Discussion section (Pages 16-17, Lines 421-435) of our revised manuscript.
3. Comment 3 – Weibull Analysis Explanation
Location: Section 3.2 – Weibull Analysis, Page 12–13, Lines 224–235
The Weibull analysis is appropriate and informative. To improve clarity for readers, please add a brief explanation on how modulus m relates to clinical reliability
**Author response:** We thank the reviewer for this helpful suggestion. To improve clarity, we have added a brief explanation of the clinical significance of the Weibull modulus (m) in both the Methods (Statistical analysis) and Results sections (Weibull analysis). Specifically, we clarify that a higher Weibull modulus indicates less variability in bond strength values and, therefore, greater consistency and predictability of bonding performance. This characteristic is clinically important because it reflects a lower likelihood of unexpected bond failures.

Please see the corresponding revisions in the updated manuscript (Methods: Page 9, Lines 240-244; Results: Page 12, Lines 286-287).
4. Comment 4 – Minor Formatting Improvements
A light editorial pass is recommended to ensure consistency in:
• Figure numbering and caption style (Figures 1–3)
• Table formatting
These edits will improve readability and professional presentation.

**Author response:** We thank the reviewer for this suggestion. We have carefully reviewed the manuscript and revised the formatting of all figures and tables to ensure consistency in numbering, caption style, and overall presentation. These changes have been made throughout the manuscript to improve readability and enhance the professional presentation of the work.

Please refer to the revised Figures and Tables in the updated manuscript.

**Reviewer #2: Dear Author**

The manuscript entitled, Bond Strength of Recycled Orthodontic Brackets After Different Reconditioning and Priming Protocols and the objective was to assess these interactions to develop evidence-based protocols for safe and effective bracket recycling, with the ultimate goal of improving clinical reliability while promoting cost efficiency and sustainability in orthodontic practice.
I have no any comments.

**Reviewer #3: Short summary**

Bond Strength of Recycled Orthodontic Brackets After Different Reconditioning and Priming Protocols reports an in‑vitro comparison of three bracket reconditioning methods (OneGloss polishing, flaming, sandblasting) combined with three primer protocols (Mani Bond, Denu Bond, none) on shear bond strength (SBS), ARI and SEM appearance. Sandblasting + primer (especially Mani Bond) produced the highest mean SBS and favorable ARI patterns; the authors conclude sandblasting is the best reconditioning method. (Manuscript PONE‑D‑25‑59612, Abstract: “Sandblasting was the most effective reconditioning method…” and “Sandblasting with Mani Bond achieved the highest SBS (9.33 ± 3.02 MPa).”)
**Major points**
========
1. Statistical reporting and methods need correction. A two‑way Welch ANOVA was used but post‑hoc testing used Tukey HSD (which assumes equal variances); replace with appropriate post‑hoc (e.g., Games‑Howell) or justify the current choice and show robustness.

**Author response:** We thank the reviewer for this important observation. In response to this comment, the statistical analyses were re-evaluated and repeated using a two-way factorial ANOVA with heteroscedasticity-robust (HC3) correction to assess the effects of bracket reconditioning method, primer protocol, and their interaction on shear bond strength (SBS). In addition, differences in SBS among the experimental groups were reanalyzed using one-way Welch ANOVA followed by Games–Howell post hoc comparisons. Consequently, the ANOVA results and associated statistical interpretations were revised. As a result of these updates, several significant patterns differed from those originally reported, and the Results, Discussion, and Conclusions have been amended accordingly to reflect the revised findings.

Please refer to the revised Methods (Page 9, Lines 236-239), Results (Page 10-11, Lines 257-265, Table 4), Discussion (Page 13-14, Lines 321-328, 329-346), and Conclusion (Page 18, Lines 461-463) sections of the revised manuscript.

2. Inconsistencies in factor degrees of freedom and labels. Table 4 lists Primer with df = 1 despite three primer levels; interaction df and other ANOVA entries appear

**Author response:** We appreciate the reviewer for identifying these inconsistencies. Following the reanalysis of the dataset, the ANOVA table has been corrected. The primer factor consisted of three levels and therefore has 2 degrees of freedom, while the interaction between adhesive removal method (3 levels) and primer application (3 levels) has 4 degrees of freedom. The revised ANOVA table now reports the correct degrees of freedom and associated test statistics. These corrections also resulted in changes to the interpretation of the findings.

Please refer to the revised Methods (Page 9, Lines 236–239), Results (Pages 10–11, Lines 257–265), and the updated Table 4 for details of these changes.
3. Experimental design ambiguity about primer/bonding agent. Methods state Denu Bond was applied as the bonding agent during initial bonding for all teeth, yet Denu Bond is also a primer level; clarify whether the primer factor duplicates the initial bonding agent and, if so, how this was controlled.

**Author response:** We thank the reviewer for this important comment and apologize for the lack of clarity in the description of the bonding procedures. During the initial bonding stage, Denu Bond was applied only to the tooth surface, while no primer was applied to the bracket base because all brackets were new. This procedure was identical for all specimens and was used solely to standardize baseline bonding conditions; therefore, it was not considered an experimental factor. Following debonding and bracket reconditioning, the experimental primer variable was introduced during the rebonding stage, with bracket bases receiving Mani Bond, Denu Bond, or no primer according to group allocation. Thus, the primer factor investigated in the present study refers exclusively to the rebonding procedure and is distinct from the standardized protocol used during initial bonding. The Methods section has been revised to clarify this distinction.

Please refer to the changes made in the Methods section (Page 7, Lines 189–195) of the revised manuscript.
4. Clinical relevance and thresholds not fully addressed. Many groups show mean SBS below commonly cited clinical thresholds (6–8 MPa). Discuss clinical acceptability explicitly and temper claims that sandblasting “restores or enhances” adhesion when most groups remain below the threshold.

**Author response:** We thank the reviewer for this important comment. We agree that the clinical relevance of in vitro shear bond strength (SBS) values should be interpreted with caution. In response to this comment, we revised the manuscript to avoid relying on a universal SBS threshold as an indicator of clinical acceptability. Instead, we emphasize that SBS values are influenced by numerous factors, including testing methodology, substrate characteristics, specimen preparation, and experimental conditions, which can contribute to substantial variability among studies [1, 2].

We further acknowledge that the relationship between in vitro bond strength measurements and clinical performance is not straightforward [3]. Therefore, the effectiveness of the rebonding protocols was interpreted primarily by comparison with the new-bracket control group tested under identical experimental conditions. This approach provides a more meaningful reference for evaluating the relative performance of the different reconditioning and primer combinations investigated in the present study.

In addition, statements suggesting that sandblasting universally “restores” or “enhances” adhesion have been revised. The Discussion and Conclusions now reflect that favorable bonding outcomes were observed only for specific combinations of reconditioning method and primer protocol.

Please refer to the revised Introduction (Page 3, Lines 87-88), Discussion (Page 15-16, Lines 383-394), and Conclusion sections (Page 18, Lines 467-468) of the updated manuscript.

5. SEM and reliability limitations. SEM sampling (two brackets per group) and Weibull moduli interpretation require caution; expand justification for SEM sample size or add more images/quantitative surface metrics (e.g., roughness, EDS) to support mechanistic claims.

**Author response:** We thank the reviewer for this valuable comment. We acknowledge that the SEM evaluation was performed on a limited number of representative specimens (two brackets per group) and was intended primarily to provide qualitative observations of surface morphology following the different reconditioning procedures. The SEM findings were not used as quantitative evidence but rather to support the interpretation of the bond strength results.

We agree that additional surface characterization methods, such as quantitative roughness measurements, energy-dispersive X-ray spectroscopy (EDS), or analysis of a larger number of specimens, would provide a more comprehensive understanding of the mechanisms underlying the observed bonding performance. However, these analyses were beyond the scope of the present study. To address this concern, we have revised the Discussion to emphasize the qualitative nature of the SEM observations and to acknowledge the lack of quantitative surface characterization as a limitation of the study.

Please refer to the changes made in the Discussion section (Page 17, Lines 443-449) of our revised manuscript.
6. Manuscript clarity and typographical errors. Multiple typos and inconsistent labels (e.g., “60” or “00” for characteristic strength, missing values in Table rows, p = 0.00) reduce credibility—correct numeric formatting, report exact p‑values or p < 0.001, and ensure all table cells are complete.

**Author response:** We thank the reviewer for this careful assessment of the manuscript. We have thoroughly reviewed the text, tables, and figures to correct typographical errors, inconsistent labels, and formatting issues. Numerical values have been checked and revised where necessary, including the correction of formatting inconsistencies in characteristic strength values (sigma zero- σ₀) and p-values. In accordance with standard reporting practices, p-values previously reported as "0.00" have been replaced with either exact values or "p < 0.001" where appropriate. We have also verified all tables to ensure that data entries are complete and consistently presented throughout the manuscript. These revisions have improved the clarity, accuracy, and overall presentation of the manuscript.

Please see the updated Table 4, 5, and Discussion section (Page 16, Line 405) of our revised manuscript.
**Minor points**
1. Power calculation detail: Provide the exact effect size, test family, and input parameters used in G*Power; report achieved power for the observed effects.

**Author response:** Thank you for your advice. We agree that adding detailed information on how the sample size was calculated would increase the study's transparency. We have revised the manuscript, adding information on how to calculate sample size with G*power (Sample size was estimated using G*Power (F tests, ANOVA: Fixed effects, omnibus, one-way) based on an effect size of f = 0.45, α = 0.05, power = 0.80, and 10 groups)

Please refer to the revised Materials and Methods section for these changes (Page 5, Lines 127-130).

2. Thermocycling rationale: State why 1,500 cycles were chosen and cite standards or precedent; discuss how this relates to clinical aging.

**Author response:** We thank the reviewer for this valuable comment. We have clarified the rationale for selecting 1,500 thermocycles in the Methods section. Previous studies have commonly used 500 thermocycles to simulate short-term aging of dental materials, while aging protocols ranging from 1,000 to 10,000 cycles have been reported depending on the study objective and material being evaluated. In the present study, 1,500 thermocycles were selected based on the protocol of Taokhampu et al. (2025), who estimated that this regimen corresponds to approximately 7 weeks of intraoral aging.

Please refer to the revised Materials and Methods section for these changes (Page 6, Lines 168-169).
3. Bonding protocol clarity: Report light‑curing irradiance in mW/cm² (1000 W/cm² is an implausible unit) and confirm curing times and distances.

**Author response:** We thank the reviewer for identifying this issue. We have corrected the unit of light-curing irradiance from W/cm² to mW/cm² and revised the bonding protocol description to provide complete details of the curing procedure, including irradiance (1000 mW/cm²), exposure time (10 s per side of the bracket; 40 s in total), and curing distance ( the light guide tip positioned as close as possible to the bracket surface, within approximately 1 mm)

Please refer to the revised Materials and Methods section (Page 6, Lines 159-164; Page 7, Line 197)
4. ARI scoring presentation: Provide a small table with counts per ARI score per group (not only figure) and include exact p‑values for the exact tests.

**Author response:** We thank the reviewer for this helpful suggestion. To improve the presentation and transparency of the ARI results, we have added a table showing the frequency distribution of each ARI score across all study groups. In addition, exact p-values from the statistical comparisons have been included in both the Results section and the table where appropriate. These revisions allow readers to more easily evaluate the distribution of failure modes among the different treatment groups.

Please refer to the updated Results section (Page 12, Line 294) and the newly added Table 6 of our revised manuscript.

5. Weibull analysis reporting: Give confidence intervals for characteristic strength and explain the method used to estimate m and characteristic strength (software, fitting method).

**Author response:** We thank the reviewer for this helpful suggestion. To improve the transparency of the Weibull analysis, we have revised the Methods and Results sections to provide additional details regarding parameter estimation. Specifically, we now state that the Weibull modulus (*m*) and characteristic strength (σ₀) were estimated by rank regression on the linearized Weibull distribution using Python (v3.12.8). In addition, 95% confidence intervals for both *m* and σ₀ have been included in the results table to facilitate interpretation of the reliability analysis.

Please refer to the changes in the Materials and Methods section (Page 9, Line 243; Page 10, Line 246) and updated Table 5 of our revised manuscript.

6. Ethics and consent: State whether written consent was obtained for use of extracted teeth and include a short sentence on donor anonymity and storage.

**Author response:** We thank the reviewer for this important comment. We have revised the Ethics statement to clarify that written informed consent was obtained from all patients whose extracted teeth were used in this study. We have also added information regarding donor anonymity and specimen handling. Specifically, all teeth were de-identified prior to collection, and no personal information was recorded. Following extraction, the teeth were stored in distilled water at room temperature until use in the experiment.

Please refer to the revised Materials and Methods section (Page 4, Line 104; Page 5, Lines 132-134) and Ethical statement section (Page 18, Lines 477-480).
7. ChatGPT use disclosure: The Acknowledgments note ChatGPT‑4o for language editing; ensure this complies with journal policy and add a short statement in Methods/Acknowledgments per journal guidance.

**Author response:** We thank the reviewer for this comment. We have revised the AI-use disclosure to align with the journal's transparency requirements. The manuscript now explicitly states that ChatGPT-4o (OpenAI, USA) was used solely for language editing and improvement of manuscript readability. All scientific content, study design, data analysis, interpretation of results, and final manuscript approval were performed by the authors. The disclosure has been included in the Acknowledgments section.

Please refer to the Acknowledgments section of the revised manuscript (Page 18, Lines 474-475).
**Concrete revision requests**
(1) Re-run statistics correcting df errors; use Welch ANOVA → Games‑Howell post‑hoc (or robust alternatives) and report effect sizes (e.g., partial 2) and exact p‑values.

**Author response:** We thank the reviewer for this valuable suggestion. Following the recommendation, we re-ran the statistical analyses, corrected the degrees-of-freedom (df) reporting, and replaced the previous post hoc procedure with the Games–Howell test, which is appropriate following Welch’s ANOVA. We also clarified the test family and input parameters used for the sample size calculation, and revised the manuscript to report exact p-values where appropriate (or p < 0.001 when applicable). These revisions improve the statistical rigor and transparency of the study

Please refer to the Methods section (Page 9, Lines 236–239), the Results section of the revised manuscript (Pages 10–11, Lines 257–265), and the updated Table 4 for details of these changes.

(2) Clarify bonding/primer workflow: explicitly state which material(s) were used as initial bonding agent and which as primer at rebonding; if the same product appears in both roles, explain controls to avoid confounding. Add a short flowchart or table of the bonding sequence for each group.

**Author response:** We thank the reviewer for this helpful suggestion. We have revised the Materials and Methods section to clearly distinguish the initial bonding and rebonding procedures. Denu Bond was used as the initial bonding agent applied to the tooth surface of all specimens to standardize baseline conditions. Following debonding and bracket reconditioning, the bracket bases received Mani Bond, Denu Bond, or no primer according to group allocation. Because the initial bonding protocol was identical across all groups, its effect was controlled and is unlikely to have confounded the comparison of rebonding protocols. A summary table of the bonding sequence has also been added for clarity.

Please refer to the changes made in the Materials and Methods section (Page 7, Lines 189-195) and Table 1 of our revised manuscript.
(3) Correct units and typos (e.g., LED irradiance, Table 3/4 labels, Weibull headings). Replace “p = 0.00” with exact values or “p < 0.001.”

**Author response:** We thank the reviewer for identifying these issues. We have corrected the LED irradiance unit to mW/cm², revised the table labels and Weibull analysis headings for consistency, and replaced instances of “p = 0.00” with “p < 0.001” where appropriate. These revisions improve the accuracy and clarity of the manuscript.

Please refer to the revised Materials and Methods section (Page 6, Line 161; Page 7, Line 197) and Table 4,5 of our revised manuscript.

(4) Expand SEM and surface analysis (or temper claims): either increase SEM sample size and add quantitative roughness/EDS data or rephrase mechanistic statements as hypotheses.

**Author response:** We thank the reviewer for this valuable suggestion. As SEM was intended as a qualitative assessment to complement the bond strength findings, we did not increase the SEM sample size or perform additional surface analyses. Instead, we have revised the manuscript to avoid overinterpreting the SEM observations and have reframed mechanistic explanations as hypotheses. We have also expanded the limitations section to acknowledge the limited SEM sample size and the absence of quantitative surface characterization.

Please refer to the expanded discussion of this limitation in the Discussion section of the revised manuscript (Page 17, Lines 443-449).

(5) Add clinical interpretation: compare group means to the 6–8 MPa clinical threshold and discuss implications for reuse in practice.

**Author response:** We thank the reviewer for this valuable suggestion. We have carefully revised the Introduction and Discussion sections to avoid citing the clinical SBS threshold of 6–8 MPa and drawing conclusions regarding bracket reuse based solely on this criterion. SBS values are influenced by multiple factors, including the testing environment, adhesive system, and experimental setup. Furthermore, bond strength results from in vitro studies may not directly reflect clinical performance. Therefore, we have emphasized comparisons with the control group tested under identical conditions, which provides a more appropriate and clinically relevant basis for interpretation.

Please refer to the revised Introduction section (Page 3, Lines 87-88) and Discussion section (Page 15-16, Lines 383-394) of our updated manuscript.

(6) Provide raw data and code (SBS values, scripts for ANOVA/Weibull) as supplementary files and include a short README so reviewers can reproduce analyses.

**Author response:** We thank the reviewer for this valuable suggestion. To enhance transparency and reproducibility, we have included the raw SBS data, statistical analysis scripts (ANOVA and Weibull analysis), and a README file describing the dataset structure and analysis workflow.

Please refer to the supplementary materials accompanying the revised manuscript.
**Recommended short text insertions and exact manuscript locations**
(1) Introduction, where sandblasting benefits are discussed (end of paragraph mentioning sandblasting):
Insert: “Recent in‑vitro work comparing enamel reconditioning methods supports sandblasting as an effective approach to restore bond strength in rebonding scenarios.”
Place after sentence: “By contrast, sandblasting has been shown in multiple studies to efficiently remove residual adhesive…”
Reference to add: Al‑Daher et al., 2024. DOI: 10.7759/cureus.66210.

**Author response:** We thank the reviewer for this helpful suggestion. We have incorporated the recommended statement into the Introduction and cited the study by Al Daher et al. (2024) to further support the use of sandblasting as an effective method for restoring bond strength in rebonding procedures.

Please refer to the revised Introduction section (Page 3, Lines 91-92).
(2) Methods, when describing sandblasting parameters (after the current parameter sentence):
Insert: “Comparable studies have evaluated sandblasting combined with acid etching for rebonding and provide methodological precedent for particle size and pressure selection.”
Place after: “Sandblasting was applied at a 90° angle to the base, 1 cm distance, 0.3 MPa pressure, for 10 s.”
Reference to add: Dirie et al., 2021. DOI: 10.1016/j.ejwf.2020.12.001.

**Author response:** We thank the reviewer for this helpful suggestion. We have added the recommended statement to the Materials and Methods section and cited the study by Dirie et al. (2021) to provide methodological support for the selected sandblasting parameters, including particle size and air pressure.

Please refer to the revised Materials and Methods section (Page 7, Lines 186-188).
(3) Introduction or Discussion, when discussing primer/adhesive systems (after paragraph on adhesive role):
Insert: “Comparative in‑vitro and in‑vivo evaluations of self‑etch and conventional primers show variable effects on bracket bond strength and should be considered when interpreting primer interactions.”
Place after: “The attachment of brackets to enamel relies not only on the mechanical retention…”
Reference to add: Alomar et al., 2024. DOI: 10.7759/cureus.72226.

**Author response:** We thank the reviewer for this helpful suggestion. We have incorporated the recommended statement into the Introduction and cited the study by Alomar et al. (2024) to provide additional context regarding the variable effects of self-etch and conventional primer systems on orthodontic bracket bond strength. This addition helps contextualize the interpretation of the primer-related findings in the present study.

Please refer to the revised Introduction section (Page 4, Lines 95-97).
(4) Methods (curing description): if LED irradiance is corrected, add a short citation about LED curing modes and bond outcomes where curing mode is discussed.
Place after curing description.
Reference to add: Najjar et al., 2023. DOI: 10.1016/j.ortho.2023.100787.

**Author response:** We thank the reviewer for this helpful suggestion. We have added the recommended citation by Najjar et al. (2023) to the Materials and Methods section following the curing protocol description. This reference provides additional support regarding the influence of LED curing parameters on orthodontic bonding outcomes.

Please refer to the revised Materials and Methods section (Page 6, Lines 162-164).

**Reviewer #4:**

**Title**
- "Bond Strength of Recycled Orthodontic Brackets After DifferentReconditioning and Priming Protocols" - first, please revise for typos.

**Author response:** We thank the reviewer for identifying this typographical error. The title has been revised accordingly, and the manuscript has been carefully proofread to correct similar typographical and formatting issues throughout the text.

Please refer to the change that has been made to the title of our revised manuscript (Page 1, Lines 1-2)
- Second, please note that you refer to bond strengths to enamel with your submitted draft. Whether you have used new or recycled brackets does not matter.

**Author response:** We thank the reviewer for this comment. We agree that the outcome measured was the bond strength of orthodontic brackets to enamel under in vitro conditions. However, this bond strength reflects the performance of the entire bracket–adhesive–enamel interface and depends on both bracket–adhesive and adhesive–enamel adhesion.

The primary aim of this study was to evaluate how bracket reconditioning methods and priming protocols affect the bonding performance of debonded orthodontic brackets. Because the experimental variables were applied to the bracket base, the focus was on how these procedures influence bracket–adhesive bonding and, consequently, the overall bond strength of the bracket–adhesive–enamel interface.

To improve clarity, we have revised the Introduction and study aim to better emphasize this objective and the clinical context of bracket rebonding.

Please refer to the changes that have been made to the Abstract section (Page 2, Line 53) and the Discussion section of our revised manuscript (Page 15, Lines 367-376)
- Third, please provide type of study with your Title.

**Author response:** We thank the reviewer for this suggestion. To improve clarity regarding the study design, we have revised the title to include the study type. The title now specifies that the investigation was conducted as an in vitro study.

Please refer to the revised title of the manuscript (Page 1, Lines 1-2)
- Finally, do not capitalize your letters. Please consult some recently published Plos One papers.

**Author response:** We thank the reviewer for this comment. The title and manuscript headings have been revised to follow the journal’s preferred capitalization style. Specifically, title capitalization has been changed to sentence case in accordance with the formatting used in recently published PLOS One articles.

Please refer to the revised title of the manuscript (Page 1, Line 1-2) and headlines of the subsections (page 4, Line 123; page 5, Line 131; page 6, Line 151; page 7, Line 174; page 8, Line 208; page 9, Line 218, 228, 234; page 10, Line 248)
**Abstract**
- Please stick to sound subheadings according to Journal style.

**Author response:** Thank you for your comment. We have revised the abstract subheadings to conform to the journal’s formatting requirements and style guidelines. We also reviewed recently published PLOS ONE articles to ensure consistency with the journal’s preferred format.

Please refer to the revised abstract in the updated manuscript (page 2, Lines 34-56).
- With your current 200 words, you do not provide maximum information. Stick to the maximum word count allowed for with this section, and revise thoroughly.

**Author response:** We thank the reviewer for this valuable comment. We have thoroughly revised the abstract to provide a more comprehensive summary of the study while remaining within the journal’s word limit (250 words). Additional details regarding the study rationale, objectives, SEM observations, ARI findings, bonding reliability, and key statistical outcomes have been incorporated to maximize the information conveyed to readers.

Please refer to the revised abstract in the updated manuscript (page 2, Lines 34-56).
- Provide sound aims and objectives.

**Author response:** Thank you for this comment. We have revised the Introduction section of the Abstract to clearly state the aim and objectives of the study. The revised abstract now explicitly specifies the investigated factors (reconditioning methods and primer protocols) and the primary outcomes evaluated (bond strength to the enamel).

Please refer to the Introduction section of our revised abstract in the updated manuscript (page 2, Lines 37-39).
- A sound rational would seem missing.

**Author response:** We thank the reviewer for this comment. We have revised the Introduction of the abstract to strengthen the rationale for the study. Specifically, we emphasize the increasing interest in rebonding debonded orthodontic brackets for economic and sustainability reasons, the importance of both bracket reconditioning and adhesive protocols in determining bond performance, and the current lack of evidence regarding their combined effects. This knowledge gap provides the rationale for the present investigation.

Please refer to the revised abstract in the updated manuscript (page 2, Lines 35-36).
- You have compared enamel bond strengths of "ninety recycled brackets", and you have compared those bond strengths to "ten new brackets", right? At the end of the day you have done a laboratory in vitro study on enamel bond strengths, no matter whether you have tested recycled or new brackets.

**Author response:** We thank the reviewer for this comment. We agree that the outcome measured was the bond strength of orthodontic brackets to enamel under in vitro conditions. However, the primary aim of this study was to evaluate how different bracket reconditioning methods and rebonding protocols influence the bonding performance of debonded orthodontic brackets. The measured bond strength reflects the performance of the entire bracket–adhesive–enamel interface and therefore depends on both the adhesion between the bracket base and the adhesive and the adhesion between the adhesive and enamel. Because the experimental variables investigated in this study (bracket reconditioning methods and priming protocols) were applied to the bracket surface and rebonding procedure, the primary objective was to determine whether these protocols could enhance the bond strength at the bracket–adhesive interface and, consequently, improve the overall bonding performance of rebonded brackets.

To improve clarity, we have also revised the Introduction, study aim, and Discussion section to emphasize that the objective of the study was to evaluate whether different bracket reconditioning methods and rebonding protocols can restore the bonding performance of debonded orthodontic brackets, using new brackets as a reference control.

Please refer to the revised Abstract (page 2, Lines 37-39), Introduction (page 4, Lines 116-121), and Discussion section (page 15, Lines 367-376) in the updated manuscript.
- You have studied (1) bracket bases with scanning electron microscopy (SEM), (2) enamel shear bond strength (SBS), and (3) (enamel) adhesive remnant index (ARI), right? Why don't you provide all results here?

**Author response:** We thank the reviewer for this comment. We agree that SEM, SBS, and ARI were all important outcome measures in this study. To provide a more balanced summary of the findings, we have revised the Results section of the Abstract to include the key SEM observations and ARI findings in addition to the SBS results.

Please refer to the updated information in the Abstract (page 2, Lines 50-53) of our revised manuscript.
- What does "m = 5.27 vs. 3.52" mean?

**Author response:** We thank the reviewer for this comment. We agree that the meaning of “m” may not be immediately clear to all readers. To improve clarity, we have revised the Abstract to explicitly define m as the Weibull modulus, a parameter that reflects the reliability and consistency of bond strength performance. In addition, we reanalyzed the Weibull data using an alternative estimation method for both the Weibull modulus and characteristic strength. Although the numerical values changed slightly, the overall trends remained unchanged.

Please refer to the revised Abstract in the updated manuscript (page 2, Line 50).
- With your Conclusions, please stick exclusively to your revised/adapted aims, and do not overstate. Do not simply repeat your results here. Do not speculate. Do not provide well-accepted (but unambiguous or even meaningless) phrases. Instead, provide a reasonable and generalizable extension of your outcome.

**Author response:** We thank the reviewer for this valuable comment. We have revised the Conclusion section of the abstract to align more closely with the study aim and to avoid overstating the findings. The revised conclusion focuses on the influence of bracket reconditioning and priming protocols on the bonding performance of debonded orthodontic brackets and highlights the broader implications of their combined effects rather than simply restating the results.

Please refer to the Conclusion section of the updated abstract in the revised manuscript (page 2, Lines 54-56.
**Introduction**

**Author response:**
- No numbers with your headlines, please. Revise thoroughly.

**Author response:** Thank you for pointing this out. We have revised the abstract and the manuscript throughout to remove numbered headings and ensure consistency with the journal’s formatting requirements.

Please refer to the revised subsection headings throughout the revised manuscript (page 4, lines 122-123; page 5, line 131; page 6, line 151; page 7, line 174; page 8, line 208; page 9, lines 218, 228, and 234; page 10, line 248).

- Revise thoroughly for sound spacebar use. Compare "23.4% [4]" and "90%[10]".

**Author response:** Thank you for this observation. We have carefully reviewed the manuscript and corrected spacing inconsistencies throughout, including the addition of spaces between text or numerical values and reference citations where appropriate, to improve the readability and consistency of the manuscript.

Please refer to the changes that have been made in our revised manuscript (page 3, Lines 71, 80).
- Please provide a sound and valid null hypothesis at the end of this section. The latter must be deducible from the forgoing thoughts.

**Author response:** Thank you for this valuable comment. We have added a null hypothesis at the end of the Introduction. The hypothesis was formulated based on the study objectives and stated that bracket reconditioning methods, primer protocols, and their interaction would have no significant effect on the bond strength of rebonded orthodontic brackets.

Please refer to the changes made at the end of the Introduction section in the revised manuscript (Page 4, Lines 119-121).
**Methods**
- Again, revise for sound headlines sticking to Journal style. Must read "Materials and methods".

**Author response:** Thank you for this comment. We have revised the section headings throughout the manuscript to ensure consistency with the journal’s formatting requirements, including changing the heading to “Materials and methods.”

Please refer to the revised subsection headings throughout the revised manuscript (page 4, lines 122-123; page 5, line 131; page 6, line 151; page 7, line 174; page 8, line 208; page 9, lines 218, 228, and 234; page 10, line 248).
- Sample size calculation must be explained in detail.

**Author response:** Thank you for this comment. We have expanded the sample size calculation section to provide complete details of the analysis, including the software used (G*Power), test family, statistical test, effect size, significance level, desired power, and the resulting minimum sample size. These additions improve the transparency and reproducibility of the study design.

Please refer to the revised Materials and methods section (Page 5, Lines 127-130).
- With ALL materials (including chemicals) and methodologies (including statistical software), please use general/non-proprietary names with your text, followed by (brand name; manufacturer, city, ST[ate - abbreviated, if US], country) in parentheses. Stick to semicolon. Please ensure reproducibility, and revise thoroughly.

**Author response:** Thank you for your valuable advices. We have thoroughly revised the Materials and methods section to ensure that all materials, chemicals, instruments, and software are reported in the journal’s recommended format, using generic names followed by the brand name, manufacturer, city, and country in parentheses and separated by semicolons where appropriate.

Please refer to the revised Materials and methods section for the updated descriptions of the materials and instruments used (Page 5, Lines 127-128, 137, 140, 141-142; page 6, Lines 153, 156-157; page 7, Lines 176-177, 179-180, 183-184, 193-194; page 8, 212-213; page 9, Line 220).

- Please add missing details to Table 1, which is not considered complete.

**Author response:** Thank you for this comment. We have revised Table 1 by adding a more detailed table legend to clarify the bonding and rebonding procedures for each group. Specifically, we now state that all specimens were initially bonded using Denu Bond, that the rebonding protocols were applied following debonding and bracket reconditioning according to group allocation, and that Group 10 consisted of new brackets serving as the control group. These additions improve the completeness and interpretability of the table.

Please refer to the revised Table 1 and its legend (pages 5-6, Lines 147-150) in the updated manuscript.
- Please avoid legal terms with your draft. Delete "™" or "GmbH & Co. KG" (and do not use "Ltd.", "®", "Inc", "CO.", "LTD.", "Corp.", "Co.", "Ltd", and so on).

**Author response:** Thank you for this comment. We have carefully reviewed the manuscript and removed trademark symbols and legal company designations (e.g., ™, ®, GmbH & Co. KG, Ltd., Inc., Corp.) where applicable. Product and manufacturer information has been revised to conform to the journal’s style requirements while maintaining sufficient detail for reproducibility.

Please refer to the changes made in our revised manuscript (page 6, Line 156; page 7, Lines 179-180).
- Details missing with "universal primer (Mani Bond (Mani, Japan)" and "5th generation primer (Denu Bond, HDI, Seoul, South Korea)". Please provide composition, and stick to main aspects like pH, viscosity, and so on.

**Author response:** Thank you for your insightful comment. We have expanded Table 2 to provide additional information on the primer materials, including their composition and relevant physicochemical properties where available. Specifically, we added the manufacturer-reported pH range for Mani Bond (mild self-etching; pH > 2, typically 2.0–2.5). Information regarding the pH of Denu Bond and the viscosity of both materials was not available from the manufacturers and is therefore indicated as “not reported by manufacturer.” These additions improve the completeness and reproducibility of the materials description.

Please refer to the revised Table 2 in the updated manuscript (page 8, Lines 202-207) .
- You have tested SBS "at the bracket–tooth interface by applying shear force". Again, this would mean that you have tested enamel bond strengths. See comments given above.

**Author response:** Thank you for your comment. We agree that the measured outcome was bond strength to enamel. However, the study specifically investigated how bracket reconditioning and priming protocols applied to the bracket base affect the overall bonding performance of debonded orthodontic brackets, which reflects both bracket–adhesive and adhesive–enamel adhesion. We have revised the Abstract, Introduction, and Discussion sections to clarify this point.

Please refer to the revised Abstract (page 2, Lines 37-39), Introduction (page 4, Lines 116-121), and Discussion section (page 15, Lines 367-376) in the updated manuscript. **Results**
- You state that "bond strengtsh ranging from 6–8 MPa are considered below the clinically acceptable range" (see Intro section). However, with the exception of Group 7, all test groups revealed lower bond strengths. These low values would seem astonishing, since you did not mention this aspect with your Abstract section.

**Author response:** We thank the reviewer for this insightful comment. We agree that most experimental groups exhibited SBS values below the 6–8 MPa clinical threshold as suggested by Reynolds et al. [4] However, SBS values are influenced by numerous factors, including testing methodology, substrate characteristics, specimen preparation, and experimental conditions, all of which can contribute to substantial variability among studies [1, 2]. In addition, the relationship between in vitro bond strength measurements and clinical performance is not straightforward [3]. Therefore, we have revised the manuscript to avoid relying on a universal SBS threshold as an indicator of clinical acceptability. We also emphasize that the clinical relevance of in vitro SBS values should be interpreted with caution and that the effectiveness of the rebonding protocols should be assessed primarily through comparison with the new-bracket control group tested under identical experimental conditions.

Please refer to the changes made in the Introduction (Page 3, Lines 87-88), Discussion (Page 15-16, Lines 383-394), and Conclusion sections (Page 18, Lines 467-468) of the updated manuscript.
- Same with "In most groups, ARI = 3 was the predominant score (ranging from 60–90% of samples)." Again, this would mean that enamel bond strengths have been tested. Same with "Groups 7-9 showing ARI scores of 0–2 accounting for 50–90% of samples".

**Author response:** We appreciate this valuable feedback. We agree that the ARI findings provide information regarding the location of bond failure within the bracket–adhesive–enamel interface. The predominance of ARI score 3 in many groups suggests that failure occurred mainly at the bracket–adhesive interface, indicating that the adhesive–enamel bond was generally stronger. Conversely, Groups 7–9 exhibited ARI scores of 0–2 in 50–90% of specimens, suggesting that the bond strength at the bracket–adhesive interface was comparable to or greater than that at the adhesive–enamel interface in a proportion of samples. These findings support our interpretation that the condition of the bracket base and the effectiveness of the reconditioning protocol contributed substantially to the overall bonding performance measured in this study. We have revised the text to clarify this interpretation.

Please refer to the changes made in the Discussion section of our updated manuscript (Page 15, Lines 373-379).
- With all Tables and Figures, please provide sound and exhausting legends. Each Table/Fig must be self-explanatory.

**Author response:** Thank you for your valuable advice. We have revised the legends of all tables and figures to provide more complete descriptions of the data presented, abbreviations used, sample sizes, statistical analyses, and other relevant information where appropriate. These revisions were made to ensure that each table and figure is self-explanatory and can be interpreted independently of the main text.

Please refer to the revised table and figure legends in the updated manuscript (page 6, Lines 148-150; page 8, Lines 203-207; page 10, Lines 251-255; page 11, Lines 267-270; page 12, Lines 280-283; page 12, Lines 296-300; page 22, Lines 646-656).
- Provide 3-digit p values. "p = 0.00" is not considered probable. Must read "p < 0.0001".

**Author response:** Thank you for this comment. We have revised the manuscript to report p-values according to the journal’s recommendations, using exact values with 3-digit where possible and reporting p < 0.001 for very small p-values.

Please refer to the changes made in the updated Table 4 of our revised manuscript (Page 11, Lines 266-270).
**Discussion**
- What about H0?

**Author response:** Thank you for this comment. We have revised the Discussion to explicitly address the study's null hypothesis. Based on the findings, the null hypothesis was partially rejected, as significant differences were observed for the primer protocol and interaction between bracket reconditioning method and primer protocol, whereas some individual main effects were not statistically significant. This statement has been added to the Discussion section.

Please refer to the changes made in the Discussion section of our updated manuscript (Page 14, Lines 326-328).
- Avoid mentioning Authors' names with your full text. Instead, stick to your main thoughts.

**Author response:** Thank you for this comment. We have revised the manuscript to reduce the use of author names in the text and instead focus on the findings and concepts being discussed, in accordance with the journal’s style. Relevant citations have been retained where appropriate.

Please refer to the Discussion sections in the updated manuscript (page 14, Lines 338; page 16, Line 394)
- Please carefully discuss aspects of enamel bonding, and the latter's influence on your outcome.

**Author response:** Thank you for your valuable suggestion. We agree that the measured bond strength reflects the performance of the entire bracket–adhesive–enamel interface and is therefore influenced by both bracket–adhesive and adhesive–enamel bonding. We have expanded the Discussion to acknowledge the contribution of enamel bonding to the measured SBS values and to clarify that enamel preparation and bonding procedures were standardized across all groups. Therefore, the observed differences are more likely attributable to the investigated bracket reconditioning and priming protocols.

Please refer to the revised Discussion sections in the updated manuscript (page 15, Lines 367-373).
- What does "highest bond strength" mean when reflecting the various ARI outcomes?

**Author response:** Thank you for this comment. We agree that bond strength and ARI evaluate different aspects of bonding performance. In our manuscript, the term “highest bond strength” refers specifically to the highest measured SBS value and does not necessarily imply the most favorable failure pattern. The ARI results provide complementary information regarding the location of bond failure within the bracket–adhesive–enamel interface. Accordingly, we have revised the text to distinguish between bond strength and failure mode and to avoid overinterpreting SBS results alone.

Please refer to the revised Discussion sections in the updated manuscript (page 15, Lines 373-379).
- Same with thoughts on "to help maintain SBS values within the optimal 6–8 MPa range". See comments given above.

**Author response:** Thank you for this comment. As noted in our response above, SBS values are influenced by numerous factors, including experimental conditions, reconditioning methods, primer systems, and adhesive materials. Therefore, the commonly cited 6–8 MPa threshold proposed by Reynolds et al.[4] should be interpreted with caution rather than as a universal criterion for clinical acceptability. In the context of the present study, comparison with the new-bracket control group tested under identical experimental conditions provides a more appropriate reference for evaluating the performance of the rebonding protocols. Accordingly, we have revised the manuscript to avoid presenting the 6–8 MPa range as an optimal clinical threshold and to provide a more balanced interpretation of the findings.

Please refer to the revised Introduction (Page 3, Lines 87-88), Discussion (Page 15-16, Lines 380-394) of the updated manuscript.

- What about the strengths of your study?

**Author response:** Thank you for this comment. We have revised the Discussion to explicitly acknowledge the strengths of the study. These include the evaluation of both bracket reconditioning methods and primer protocols within the same experimental design, allowing assessment of their individual and combined effects on bonding performance. In addition, the use of complementary outcome measures, including Weibull analysis alongside conventional bond strength testing, provided a more comprehensive evaluation of bonding performance and reliability.

Please refer to the revised Discussion section (Page 17, Lines 436-442).
- What about the responses to your teaching objectives?

**Author response:** Thank you for this comment. We have revised the Results and Discussion sections to more clearly relate the findings to the study objectives. Specifically, we highlight how the results address the effects of bracket reconditioning methods and primer protocols on bond strength, bonding reliability, and failure mode, thereby ensuring that each study objective is directly addressed by the presented findings.

Please refer to the revised Discussion section (page 13, Lines 321-324; page 14, Lines 329-330; page 15, Lines 380-382).
**Conclusion**
- Again, with your Conclusions, please stick exclusively to your revised/adapted aims, and do not overstate. Do not simply repeat your results here. Do not speculate. Do not provide well-accepted (but unambiguous or even meaningless) phrases. Instead, provide a reasonable and generalizable extension of your outcome. Remember that "Conclusion" does neither mean Repetition of Results" nor "Summary".

**Author response:** Thank you for this constructive feedback. We have revised the Conclusion to align more closely with the study aims and to avoid overstatement. The revised conclusion focuses on the overall implications of the interaction between bracket reconditioning and primer protocols for the bonding performance of rebonded orthodontic brackets, rather than repeating specific results or making broad clinical claims.

Please refer to the revised Conclusion section in the updated manuscript (page 18, Lines 461-463, 467-468)
**Refs**
- References have not been adapted to sound and uniform Journal style.
In total, several shortcomings do not allow for any proceeding with this submitted draft. Many aspects would call for a sound and thorough clarification.

**Author response:** Thank you for this observation. We have thoroughly reviewed and revised the references to ensure that they conform to the journal’s style and are presented in a consistent format throughout the manuscript.

Please refer to the changes made to the reference section in the revised manuscript (Page 19-23, Lines 494–644).

**References**

1. Ferracane JL, Hilton TJ, and Sakaguchi RL. Introduction to and outcomes of the conference on adhesion in dentistry*.* Dent Mater. 2010; 26 (2): 105-7.

2. Marshall SJ, Bayne SC, Baier R, Tomsia AP, and Marshall GW. A review of adhesion science*.* Dent Mater. 2010; 26 (2): e11-6.

3. De Munck J, Van Landuyt K, Peumans M, Poitevin A, Lambrechts P, Braem M, et al. A critical review of the durability of adhesion to tooth tissue: methods and results*.* J Dent Res. 2005; 84 (2): 118-32.

4. Reynolds I. A review of direct orthodontic bonding*.* British journal of orthodontics. 1975; 2 (3): 171-178.
