# Supplementary material for: Bond strength of debonded orthodontic brackets after different reconditioning and priming protocols: An in vitro study
Source: PLoS One. 2026 Jul 7;21(7):e0353265. doi: 10.1371/journal.pone.0353265 (PMC13340843; doi:10.1371/journal.pone.0353265)
Supplement: Peer review history

# Peer review: Decision Letter 0

**DOI:** 10.1371/journal.pone.0353265.r001

**Authors:** Andrej Kielbassa

8 Apr 2026

PONE-D-25-59612

Bond Strength of Recycled Orthodontic Brackets After Different Reconditioning and Priming Protocols

PLOS One

Dear Dr. Nguyen,

Thank you for submitting your manuscript to PLOS ONE. After careful consideration, we feel that it has merit but does not fully meet PLOS ONE’s publication criteria as it currently stands. Therefore, we invite you to submit a revised version of the manuscript that addresses the points raised during the review process.

First, please note that the previous Editor has stepped back from his tasks, and I have been invited to take over your submitted draft. Please accept our apologies for the time having passed since your submission.

- While differing with their recommendations to some some extent, our external reviewers have finally addressed several shortcomings and drawbacks, and the latter have been indicated below.
- I have additionally double checked your submitted draft, to come to a more balanced decision. Specific feedback from my evaluation of the manuscript has been added below. You surely will agree that your manuscript is not considered ready to proceed.
- Please note that I have decided to give you the opportunity to carefully but thoroughly revise your draft. You should bear in mind that our final decision will depend on the quality of your revised draft, and that one revision is considered standard. Remember that re-review of your re-submitted draft is considered mandatory.

Please submit your revised manuscript by May 23 2026 11:59PM. If you will need more time than this to complete your revisions, please reply to this message or contact the journal office at plosone@plos.org. When you're ready to submit your revision, log on to https://www.editorialmanager.com/pone/ and select the 'Submissions Needing Revision' folder to locate your manuscript file.

Please include the following items when submitting your revised manuscript:

- A letter that responds to each point raised by the academic editor and reviewer(s). You should upload this letter as a separate file labeled 'Response to Reviewers'.
- A marked-up copy of your manuscript that highlights changes made to the original version. You should upload this as a separate file labeled 'Revised Manuscript with Track Changes'.
- An unmarked version of your revised paper without tracked changes. You should upload this as a separate file labeled 'Manuscript'.

If you would like to make changes to your financial disclosure, please include your updated statement in your cover letter. Guidelines for resubmitting your figure files are available below the reviewer comments at the end of this letter.

If applicable, we recommend that you deposit your laboratory protocols in protocols.io to enhance the reproducibility of your results. Protocols.io assigns your protocol its own identifier (DOI) so that it can be cited independently in the future. For instructions see: https://journals.plos.org/plosone/s/submission-guidelines#loc-laboratory-protocols. Additionally, PLOS ONE offers an option for publishing peer-reviewed Lab Protocol articles, which describe protocols hosted on protocols.io. Read more information on sharing protocols at https://plos.org/protocols?utm_medium=editorial-email&utm_source=authorletters&utm_campaign=protocols.

As the corresponding author, your ORCID iD is verified in the submission system and will appear in the published article. PLOS supports the use of ORCID, and we encourage all coauthors to register for an ORCID iD and use it as well. Please encourage your coauthors to verify their ORCID iD within the submission system before final acceptance, as unverified ORCID iDs will not appear in the published article. *Only*  the individual author can complete the verification step; PLOS staff *cannot*  verify ORCID iDs on behalf of authors.

We look forward to receiving your revised manuscript.

Kind regards,

Prof. Dr. Dr. h. c. Andrej M Kielbassa

Academic Editor

PLOS One

Journal Requirements:

When submitting your revision, we need you to address these additional requirements.

1. Please ensure that your manuscript meets PLOS ONE's style requirements, including those for file naming. The PLOS ONE style templates can be found at

https://journals.plos.org/plosone/s/file?id=wjVg/PLOSOne_formatting_sample_main_body.pdf and https://journals.plos.org/plosone/s/file?id=ba62/PLOSOne_formatting_sample_title_authors_affiliations.pdf

2. Please include captions for your Supporting Information files at the end of your manuscript, and update any in-text citations to match accordingly. Please see our Supporting Information guidelines for more information: http://journals.plos.org/plosone/s/supporting-information.

If the reviewer comments include a recommendation to cite specific previously published works, please review and evaluate these publications to determine whether they are relevant and should be cited. There is no requirement to cite these works unless the editor has indicated otherwise.

[Note: HTML markup is below. Please do not edit.]

Reviewers' comments:

Reviewer's Responses to Questions

**Comments to the Author**

1. Is the manuscript technically sound, and do the data support the conclusions?

The manuscript must describe a technically sound piece of scientific research with data that supports the conclusions. Experiments must have been conducted rigorously, with appropriate controls, replication, and sample sizes. The conclusions must be drawn appropriately based on the data presented.

Reviewer #1: Yes

Reviewer #2: Yes

Reviewer #3: Partly

Reviewer #4: No

**********

2. Has the statistical analysis been performed appropriately and rigorously?

Reviewer #1: Yes

Reviewer #2: Yes

Reviewer #3: Yes

Reviewer #4: No

**********

3. Have the authors made all data underlying the findings in their manuscript fully available?

The PLOS Data policy requires authors to make all data underlying the findings described in their manuscript fully available without restriction, with rare exception (please refer to the Data Availability Statement in the manuscript PDF file). The data should be provided as part of the manuscript or its supporting information, or deposited to a public repository. For example, in addition to summary statistics, the data points behind means, medians and variance measures should be available. If there are restrictions on publicly sharing data—e.g. participant privacy or use of data from a third party—those must be specified.

Reviewer #1: Yes

Reviewer #2: Yes

Reviewer #3: No

Reviewer #4: No

**********

4. Is the manuscript presented in an intelligible fashion and written in standard English?

PLOS ONE does not copyedit accepted manuscripts, so the language in submitted articles must be clear, correct, and unambiguous. Any typographical or grammatical errors should be corrected at revision, so please note any specific errors here.

Reviewer #1: Yes

Reviewer #2: Yes

Reviewer #3: Yes

Reviewer #4: Yes

**********

5. Review Comments to the Author

Please use the space provided to explain your answers to the questions above. You may also include additional comments for the author, including concerns about dual publication, research ethics, or publication ethics. (Please upload your review as an attachment if it exceeds 20,000 characters)

Reviewer #1: Comments for the Authors

Comment 1 – Suggest adding some definitions and importance of performance indicator parameters (i.e - SBS, ARI, bonding reliability etc), this could make the manuscript more coherent and user-friendly for broader audience.

Comment 2 – Discussion of Potential Enamel Risk with High SBS Groups

Location: Section 4, Page 20, Lines 337–340

While mentioned briefly in the Discussion, please expand on the clinical implications regarding enamel crack risk during debonding, and the balance between strong adhesion and safe removal procedures.

Comment 3 – Weibull Analysis Explanation

Location: Section 3.2 – Weibull Analysis, Page 12–13, Lines 224–235

The Weibull analysis is appropriate and informative. To improve clarity for readers, please add a brief explanation on how modulus m relates to clinical reliability

Comment 4 – Minor Formatting Improvements

A light editorial pass is recommended to ensure consistency in:

• Figure numbering and caption style (Figures 1–3)

• Table formatting

These edits will improve readability and professional presentation.

Reviewer #2: Dear Author,

The manuscript entitled, Bond Strength of Recycled Orthodontic Brackets After Different Reconditioning and Priming Protocols and the objective was to assess these interactions to develop evidence-based protocols for safe and effective bracket recycling, with the ultimate goal of improving clinical reliability while promoting cost efficiency and sustainability in orthodontic practice.

I have no any comments.

Reviewer #3: Short summary

===========

Bond Strength of Recycled Orthodontic Brackets After Different Reconditioning and Priming Protocols reports an in‑vitro comparison of three bracket reconditioning methods (OneGloss polishing, flaming, sandblasting) combined with three primer protocols (Mani Bond, Denu Bond, none) on shear bond strength (SBS), ARI and SEM appearance. Sandblasting + primer (especially Mani Bond) produced the highest mean SBS and favorable ARI patterns; the authors conclude sandblasting is the best reconditioning method. (Manuscript PONE‑D‑25‑59612, Abstract: “Sandblasting was the most effective reconditioning method…” and “Sandblasting with Mani Bond achieved the highest SBS (9.33 ± 3.02 MPa).”)

Major points

========

(1) Statistical reporting and methods need correction. A two‑way Welch ANOVA was used but post‑hoc testing used Tukey HSD (which assumes equal variances); replace with appropriate post‑hoc (e.g., Games‑Howell) or justify the current choice and show robustness.

(2) Inconsistencies in factor degrees of freedom and labels. Table 4 lists Primer with df = 1 despite three primer levels; interaction df and other ANOVA entries appear inconsistent—these must be corrected and rechecked in the analysis code and tables.

(3) Experimental design ambiguity about primer/bonding agent. Methods state Denu Bond was applied as the bonding agent during initial bonding for all teeth, yet Denu Bond is also a primer level; clarify whether the primer factor duplicates the initial bonding agent and, if so, how this was controlled.

(4) Clinical relevance and thresholds not fully addressed. Many groups show mean SBS below commonly cited clinical thresholds (6–8 MPa). Discuss clinical acceptability explicitly and temper claims that sandblasting “restores or enhances” adhesion when most groups remain below the threshold.

(5) SEM and reliability limitations. SEM sampling (two brackets per group) and Weibull moduli interpretation require caution; expand justification for SEM sample size or add more images/quantitative surface metrics (e.g., roughness, EDS) to support mechanistic claims.

(6) Manuscript clarity and typographical errors. Multiple typos and inconsistent labels (e.g., “60” or “00” for characteristic strength, missing values in Table rows, p = 0.00) reduce credibility—correct numeric formatting, report exact p‑values or p < 0.001, and ensure all table cells are complete.

Minor points

========

(1) Power calculation detail: Provide the exact effect size, test family, and input parameters used in G*Power; report achieved power for the observed effects.

(2) Thermocycling rationale: State why 1,500 cycles were chosen and cite standards or precedent; discuss how this relates to clinical aging.

(3) Bonding protocol clarity: Report light‑curing irradiance in mW/cm² (1000 W/cm² is implausible unit) and confirm curing times and distances.

(4) ARI scoring presentation: Provide a small table with counts per ARI score per group (not only figure) and include exact p‑values for the exact tests.

(5) Weibull analysis reporting: Give confidence intervals for characteristic strength and explain the method used to estimate m and characteristic strength (software, fitting method).

(6) Ethics and consent: State whether written consent was obtained for use of extracted teeth and include a short sentence on donor anonymity and storage.

(6) ChatGPT use disclosure: The Acknowledgments note ChatGPT‑4o for language editing; ensure this complies with journal policy and add a short statement in Methods/Acknowledgments per journal guidance.

Concrete revision requests

=================

(1) Re-run statistics correcting df errors; use Welch ANOVA → Games‑Howell post‑hoc (or robust alternatives) and report effect sizes (e.g., partial 2) and exact p‑values.

(2) Clarify bonding/primer workflow: explicitly state which material(s) were used as initial bonding agent and which as primer at rebonding; if the same product appears in both roles, explain controls to avoid confounding. Add a short flowchart or table of the bonding sequence for each group.

(3) Correct units and typos (e.g., LED irradiance, Table 3/4 labels, Weibull headings). Replace “p = 0.00” with exact values or “p < 0.001.”

(4) Expand SEM and surface analysis (or temper claims): either increase SEM sample size and add quantitative roughness/EDS data or rephrase mechanistic statements as hypotheses.

(5) Add clinical interpretation: compare group means to the 6–8 MPa clinical threshold and discuss implications for reuse in practice.

(6) Provide raw data and code (SBS values, scripts for ANOVA/Weibull) as supplementary files and include a short README so reviewers can reproduce analyses.

Recommended short text insertions and exact manuscript locations

=========================================

(1) Introduction, where sandblasting benefits are discussed (end of paragraph mentioning sandblasting):

Insert: “Recent in‑vitro work comparing enamel reconditioning methods supports sandblasting as an effective approach to restore bond strength in rebonding scenarios.”

Place after sentence: “By contrast, sandblasting has been shown in multiple studies to efficiently remove residual adhesive…”

Reference to add: Al‑Daher et al., 2024. DOI: 10.7759/cureus.66210.

(2) Methods, when describing sandblasting parameters (after the current parameter sentence):

Insert: “Comparable studies have evaluated sandblasting combined with acid etching for rebonding and provide methodological precedent for particle size and pressure selection.”

Place after: “Sandblasting was applied at a 90° angle to the base, 1 cm distance, 0.3 MPa pressure, for 10 s.”

Reference to add: Dirie et al., 2021. DOI: 10.1016/j.ejwf.2020.12.001.

(3) Introduction or Discussion, when discussing primer/adhesive systems (after paragraph on adhesive role):

Insert: “Comparative in‑vitro and in‑vivo evaluations of self‑etch and conventional primers show variable effects on bracket bond strength and should be considered when interpreting primer interactions.”

Place after: “The attachment of brackets to enamel relies not only on the mechanical retention…”

Reference to add: Alomar et al., 2024. DOI: 10.7759/cureus.72226.

(4) Methods (curing description): if LED irradiance is corrected, add a short citation about LED curing modes and bond outcomes where curing mode is discussed.

Place after curing description.

Reference to add: Najjar et al., 2023. DOI: 10.1016/j.ortho.2023.100787.

Reviewer #4: Title

- "Bond Strength of Recycled Orthodontic Brackets After DifferentReconditioning and Priming Protocols" - first, please revise for typos.

- Second, please note that you refer to bond strengths to enamel with your submitted draft. Whether you have used new or recycled brackets does not matter.

- Third, please provide type of study with your Title.

- Finally, do not capitalize your letters. Please consult some recently published Plos One papers.

Abstract

- Please stick to sound subheadings according to Journal style.

- With your current 200 words, you do not provide maximum information. Stick to the maximum word count allowed for with this section, and revise thoroughly.

- Provide sound aims and objectives.

- A sound rational would seem missing.

- You have compared enamel bond strengths of "ninety recycled brackets", and you have compared those bond strengths to "ten new brackets", right? At the end of the day you have done a laboratory in vitro study on enamel bond strengths, no matter whether you have tested recycled or new brackets.

- You have studied (1) bracket bases with scanning electron microscopy (SEM), (2) enamel shear bond strength (SBS), and (3) (enamel) adhesive remnant index (ARI), right? Why don't you provide all results here?

- What does "m = 5.27 vs. 3.52" mean?

- With your Conclusions, please stick exclusively to your revised/adapted aims, and do not overstate. Do not simply repeat your results here. Do not speculate. Do not provide well-accepted (but unambiguous or even meaningless) phrases. Instead, provide a reasonable and generalizable extension of your outcome.

Intro

- No numbers with your headlines, please. Revise thoroughly.

- Revise thoroughly for sound spacebar use. Compare "23.4% [4]" and "90%[10]".

- Please provide a sound and valid null hypothesis at the end of this section. The latter must be deducible from the forgoing thoughts.

Methods

- Again, revise for sound headlines sticking to Journal style. Must read "Materials and methods".

- Sample size calculation must be explained in detail.

- With ALL materials (including chemicals) and methodologies (including statistical software), please use general/non-proprietary names with your text, followed by (brand name; manufacturer, city, ST[ate - abbreviated, if US], country) in parentheses. Stick to semicolon. Please ensure reproducibility, and revise thoroughly.

- Please add missing details to Table 1, which is not considered complete.

- Please avoid legal terms with your draft. Delete "™" or "GmbH & Co. KG" (and do not use "Ltd.", "®", "Inc", "CO.", "LTD.", "Corp.", "Co.", "Ltd", and so on).

- Details missing with "universal primer (Mani Bond (Mani, Japan)" and "5th generation primer (Denu Bond, HDI, Seoul, South Korea)". Please provide composition, and stick to main aspects like pH, viscosity, and so on.

- You have tested SBS "at the bracket–tooth interface by applying shear force". Again, this would mean that you have tested enamel bond strengths. See comments given above.

Results

- You state that "bond strengtsh ranging from 6–8 MPa are considered below the clinically acceptable range" (see Intro section). However, with the exception of Group 7, all test groups revealed lower bond strengths. These low values would seem astonishing, since you did not mention this aspect with your Abstract section.

- Same with "In most groups, ARI = 3 was the predominant score (ranging from 60–90% of samples)." Again, this would mean that enamel bond strengths have been tested.

- Same with "Groups 7-9 showing ARI scores of 0–2 accounting for 50–90% of samples".

- With all Tables and Figures, please provide sound and exhausting legends. Each Table/Fig must be self-explanatory.

- Provide 3-digit p values.

- "p = 0.00" is not considered probable. Must read "p < 0.0001".

Disc

- What about H0?

- Avoid mentioning Authors' names with your full text. Instead, stick to your main thoughts.

- Please carefully discuss aspects of enamel bonding, and the latter's influence on your outcome.

- What does "highest bond strength" mean when reflecting the various ARI outcomes?

- Same with thoughts on "to help maintain SBS values within the optimal 6–8 MPa range". See comments given above.

- What about the strengths of your study?

- What about the responses to your teaching objectives?

Concl

- Again, with your Conclusions, please stick exclusively to your revised/adapted aims, and do not overstate. Do not simply repeat your results here. Do not speculate. Do not provide well-accepted (but unambiguous or even meaningless) phrases. Instead, provide a reasonable and generalizable extension of your outcome.

- Remember that "Conclusion" does neither mean Repetition of Results" nor "Summary".

Refs

- References have not been adapted to sound and uniform Journal style.

In total, several shortcomings do not allow for any proceeding with this submitted draft. Many aspects would call for a sound and thorough clarification.

**********

6. PLOS authors have the option to publish the peer review history of their article (what does this mean?). If published, this will include your full peer review and any attached files.

If you choose “no”, your identity will remain anonymous but your review may still be made public.

**Do you want your identity to be public for this peer review?**  For information about this choice, including consent withdrawal, please see our Privacy Policy.

Reviewer #1: No

Reviewer #2: No

Reviewer #3: No

Reviewer #4: No

**********

[NOTE: If reviewer comments were submitted as an attachment file, they will be attached to this email and accessible via the submission site. Please log into your account, locate the manuscript record, and check for the action link "View Attachments". If this link does not appear, there are no attachment files.]

To ensure your figures meet our technical requirements, please review our figure guidelines: https://journals.plos.org/plosone/s/figures

You may also use PLOS’s free figure tool, NAAS, to help you prepare publication quality figures: https://journals.plos.org/plosone/s/figures#loc-tools-for-figure-preparation.

NAAS will assess whether your figures meet our technical requirements by comparing each figure against our figure specifications.

Attachment Submitted filename: Reviewer_Report_PONE-D-25-59612_Bond_Strength.docx

# Author comment: Author response to Decision Letter 1

**DOI:** 10.1371/journal.pone.0353265.r002

15 Jun 2026

Manuscript ID. PONE-D-25-59612

Response to Reviewers

Dear Editorial Board

PLOS ONE

Thank you for giving us the opportunity to submit a revised draft of the manuscript “Bond Strength of Recycled Orthodontic Brackets After Different Reconditioning and Priming Protocols” for publication in the PLOS ONE. We appreciate the time and effort you and the reviewers dedicated to providing feedback on our manuscript and are grateful for the insightful comments and valuable improvements to our paper.

We have incorporated most of the suggestions made by the reviewers. Those changes are highlighted within the manuscript in red. Please see below, in blue, for a point-by-point response to the reviewers’ comments and concerns. All page numbers refer to the revised manuscript file with tracked changes.

Reviewer #1: Comments for the Authors

1. Comment 1 – Suggest adding some definitions and importance of performance indicator parameters (i.e - SBS, ARI, bonding reliability etc), this could make the manuscript more coherent and user-friendly for broader audience.

Author response: We thank the reviewer for this helpful suggestion. To improve clarity and accessibility for a broader readership, we have added brief definitions and clinical relevance of key performance indicators, including shear bond strength (SBS), adhesive remnant index (ARI), and bonding reliability (Weibull analysis), in the Introduction section. These additions aim to enhance the coherence of the manuscript and facilitate interpretation of the results.

Please refer to the corresponding revisions in the manuscript (page 4, lines 101-110)

2. Comment 2 – Discussion of Potential Enamel Risk with High SBS Groups

Location: Section 4, Page 20, Lines 337–340

While mentioned briefly in the Discussion, please expand on the clinical implications regarding enamel crack risk during debonding, and the balance between strong adhesion and safe removal procedures.

Author response: We thank the reviewer for this valuable suggestion. We agree that the clinical implications of excessively high bond strength warrant further discussion. Accordingly, we have expanded the Discussion section to address the balance between achieving sufficient bond strength for clinical performance and avoiding excessively high bond strength that may increase the risk of enamel damage during bracket removal. We now discuss the potential for enamel cracks associated with high debonding forces, the importance of selecting bonding protocols that provide adequate but not excessive adhesion.

Please refer to the revised text that has been added to the Discussion section (Pages 16-17, Lines 421-435) of our revised manuscript.

3. Comment 3 – Weibull Analysis Explanation

Location: Section 3.2 – Weibull Analysis, Page 12–13, Lines 224–235

The Weibull analysis is appropriate and informative. To improve clarity for readers, please add a brief explanation on how modulus m relates to clinical reliability

Author response: We thank the reviewer for this helpful suggestion. To improve clarity, we have added a brief explanation of the clinical significance of the Weibull modulus (m) in both the Methods (Statistical analysis) and Results sections (Weibull analysis). Specifically, we clarify that a higher Weibull modulus indicates less variability in bond strength values and, therefore, greater consistency and predictability of bonding performance. This characteristic is clinically important because it reflects a lower likelihood of unexpected bond failures.

Please see the corresponding revisions in the updated manuscript (Methods: Page 9, Lines 240-244; Results: Page 12, Lines 286-287).

4. Comment 4 – Minor Formatting Improvements

A light editorial pass is recommended to ensure consistency in:

• Figure numbering and caption style (Figures 1–3)

• Table formatting

These edits will improve readability and professional presentation.

Author response: We thank the reviewer for this suggestion. We have carefully reviewed the manuscript and revised the formatting of all figures and tables to ensure consistency in numbering, caption style, and overall presentation. These changes have been made throughout the manuscript to improve readability and enhance the professional presentation of the work.

Please refer to the revised Figures and Tables in the updated manuscript.

Reviewer #2: Dear Author

The manuscript entitled, Bond Strength of Recycled Orthodontic Brackets After Different Reconditioning and Priming Protocols and the objective was to assess these interactions to develop evidence-based protocols for safe and effective bracket recycling, with the ultimate goal of improving clinical reliability while promoting cost efficiency and sustainability in orthodontic practice.

I have no any comments.

Reviewer #3: Short summary

Bond Strength of Recycled Orthodontic Brackets After Different Reconditioning and Priming Protocols reports an in vitro comparison of three bracket reconditioning methods (OneGloss polishing, flaming, sandblasting) combined with three primer protocols (Mani Bond, Denu Bond, none) on shear bond strength (SBS), ARI and SEM appearance. Sandblasting + primer (especially Mani Bond) produced the highest mean SBS and favorable ARI patterns; the authors conclude sandblasting is the best reconditioning method. (Manuscript PONE D 25 59612, Abstract: “Sandblasting was the most effective reconditioning method…” and “Sandblasting with Mani Bond achieved the highest SBS (9.33 ± 3.02 MPa).”)

Major points

========

1. Statistical reporting and methods need correction. A two way Welch ANOVA was used but post hoc testing used Tukey HSD (which assumes equal variances); replace with appropriate post hoc (e.g., Games Howell) or justify the current choice and show robustness.

Author response: We thank the reviewer for this important observation. In response to this comment, the statistical analyses were re-evaluated and repeated using a two-way factorial ANOVA with heteroscedasticity-robust (HC3) correction to assess the effects of bracket reconditioning method, primer protocol, and their interaction on shear bond strength (SBS). In addition, differences in SBS among the experimental groups were reanalyzed using one-way Welch ANOVA followed by Games–Howell post hoc comparisons. Consequently, the ANOVA results and associated statistical interpretations were revised. As a result of these updates, several significant patterns differed from those originally reported, and the Results, Discussion, and Conclusions have been amended accordingly to reflect the revised findings.

Please refer to the revised Methods (Page 9, Lines 236-239), Results (Page 10-11, Lines 257-265, Table 4), Discussion (Page 13-14, Lines 321-328, 329-346), and Conclusion (Page 18, Lines 461-463) sections of the revised manuscript.

2. Inconsistencies in factor degrees of freedom and labels. Table 4 lists Primer with df = 1 despite three primer levels; interaction df and other ANOVA entries appear

Author response: We appreciate the reviewer for identifying these inconsistencies. Following the reanalysis of the dataset, the ANOVA table has been corrected. The primer factor consisted of three levels and therefore has 2 degrees of freedom, while the interaction between adhesive removal method (3 levels) and primer application (3 levels) has 4 degrees of freedom. The revised ANOVA table now reports the correct degrees of freedom and associated test statistics. These corrections also resulted in changes to the interpretation of the findings.

Please refer to the revised Methods (Page 9, Lines 236–239), Results (Pages 10–11, Lines 257–265), and the updated Table 4 for details of these changes.

3. Experimental design ambiguity about primer/bonding agent. Methods state Denu Bond was applied as the bonding agent during initial bonding for all teeth, yet Denu Bond is also a primer level; clarify whether the primer factor duplicates the initial bonding agent and, if so, how this was controlled.

Author response: We thank the reviewer for this important comment and apologize for the lack of clarity in the description of the bonding procedures. During the initial bonding stage, Denu Bond was applied only to the tooth surface, while no primer was applied to the bracket base because all brackets were new. This procedure was identical for all specimens and was used solely to standardize baseline bonding conditions; therefore, it was not considered an experimental factor. Following debonding and bracket reconditioning, the experimental primer variable was introduced during the rebonding stage, with bracket bases receiving Mani Bond, Denu Bond, or no primer according to group allocation. Thus, the primer factor investigated in the present study refers exclusively to the rebonding procedure and is distinct from the standardized protocol used during initial bonding. The Methods section has been revised to clarify this distinction.

Please refer to the changes made in the Methods section (Page 7, Lines 189–195) of the revised manuscript.

4. Clinical relevance and thresholds not fully addressed. Many groups show mean SBS below commonly cited clinical thresholds (6–8 MPa). Discuss clinical acceptability explicitly and temper claims that sandblasting “restores or enhances” adhesion when most groups remain below the threshold.

Author response: We thank the reviewer for this important comment. We agree that the clinical relevance of in vitro shear bond strength (SBS) values should be interpreted with caution. In response to this comment, we revised the manuscript to avoid relying on a universal SBS threshold as an indicator of clinical acceptability. Instead, we emphasize that SBS values are influenced by numerous factors, including testing methodology, substrate characteristics, specimen preparation, and experimental conditions, which can contribute to substantial variability among studies [1, 2].

We further acknowledge that the relationship between in vitro bond strength measurements and clinical performance is not straightforward [3]. Therefore, the effectiveness of the rebonding protocols was interpreted primarily by comparison with the new-bracket control group tested under identical experimental conditions. This approach provides a more meaningful reference for evaluating the relative performance of the different reconditioning and primer combinations investigated in the present study.

In addition, statements suggesting that sandblasting universally “restores” or “enhances” adhesion have been revised. The Discussion and Conclusions now reflect that favorable bonding outcomes were observed only for specific combinations of reconditioning method and primer protocol.

Please refer to the revised Introduction (Page 3, Lines 87-88), Discussion (Page 15-16, Lines 383-394), and Conclusion sections (Page 18, Lines 467-468) of the updated manuscript.

5. SEM and reliability limitations. SEM sampling (two brackets per group) and Weibull moduli interpretation require caution; expand justification for SEM sample size or add more images/quantitative surface metrics (e.g., roughness, EDS) to support mechanistic claims.

Author response: We thank the reviewer for this valuable comment. We acknowledge that the SEM evaluation was performed on a limited number of representative specimens (two brackets per group) and was intended primarily to provide qualitative observations of surface morphology following the different reconditioning procedures. The SEM findings were not used as quantitative evidence but rather to support the interpretation of the bond strength results.

We agree that additional surface characterization methods, such as quantitative roughness measurements, energy-dispersive X-ray spectroscopy (EDS), or analysis of a larger number of specimens, would provide a more comprehensive understanding of the mechanisms underlying the observed bonding performance. However, these analyses were beyond the scope of the present study. To address this concern, we have revised the Discussion to emphasize the qualitative nature of the SEM observations and to acknowledge the lack of quantitative surface characterization as a limitation of the study.

Please refer to the changes made in the Discussion section (Page 17, Lines 443-449) of our revised manuscript.

6. Manuscript clarity and typographical errors. Multiple typos and inconsistent labels (e.g., “60” or “00” for characteristic strength, missing values in Table rows, p = 0.00) reduce credibility—correct numeric formatting, report exact p values or p < 0.001, and ensure all table cells are complete.

Author response: We thank the reviewer for this careful assessment of the manuscript. We have thoroughly reviewed the text, tables, and figures to correct typographical errors, inconsistent labels, and formatting issues. Numerical values have been checked and revised where necessary, including the correction of formatting inconsistencies in characteristic strength values (sigma zero- σ₀) and p-values. In accordance with standard reporting practices, p-values previously reported as "0.00" have been replaced with either exact values or "p < 0.001" where appropriate. We have also verified all tables to ensure that data entries are complete and consistently presented throughout the manuscript. These revisions have improved the clarity, accuracy, and overall presentation of the manuscript.

Please see the updated Table 4, 5, and Discussion section (Page 16, Line 405) of our revised manuscript.

Minor points

1. Power calculation detail: Provide the exact effect size, test family, and input parameters used in G*Power; report achieved power for the observed effects.

Author response: Thank you for your advice. We agree that adding detailed information on how the sample size was calculated would increase the study's transparency. We have revised the manuscript, adding information on how to calculate sample size with G*power (Sample size was estimated using G*Power (F tests, ANOVA: Fixed effects, omnibus, one-way) based on an effect size of f = 0.45, α = 0.05, power = 0.80, and 10 groups)

Please refer to the revised Materials and Methods section for these changes (Page 5, Lines 127-130).

2. Thermocycling rationale: State why 1,500 cycles were chosen and cite standards or precedent; discuss how this relates to clinical aging.

Author response: We thank the reviewer for this valuable comment. We have clarified the rationale for selecting 1,500 thermocycles in the Methods section. Previous studies have commonly used 500 thermocycles to simulate short-term aging of dental materials, while aging protocols ranging from 1,000 to 10,000 cycles have been reported depending on the study objective and material being evaluated. In the present study, 1,500 thermocycles were selected based on the protocol of Taokhampu et al. (2025), who estimated that this regimen corresponds to approximately 7 weeks of intraoral aging.

Please refer to the revised Materials and Methods section for these changes (Page 6, Lines 168-169).

3. Bonding protocol clarity: Report light curing irradiance in mW/cm² (1000 W/cm² is an implausible unit) and confirm curing times and distances.

Author response: We thank the reviewer for identifying this issue. We have corrected the unit of light-curing irradiance from W/cm² to mW/cm² and revised the bonding protocol description to provide complete details of the curing procedure, including irradiance (1000 mW/cm²), exposure time (10 s per side of the bracket; 40 s in total), and curing distance ( the light guide tip positioned as close as possible to the bracket surface, within approximately 1 mm)

Please refer to the revised Materials and Methods section (Page 6, Lines 159-164; Page 7, Line 197)

4. ARI scoring presentation: Provide a small table with counts per ARI score per group (not only figure) and include exact p values for the exact tests.

Author response: We thank the reviewer for this helpful suggestion. To improve the presentation and transparency of the ARI results, we have added a table showing the frequency distribution of each ARI score across all study groups. In addition, exact p-values from the statistical comparisons have been included in both the Results section and the table where appropriate. These revisions allo

Attachment Submitted filename: Response to Reviewers.docx

# Peer review: Decision Letter 1

**DOI:** 10.1371/journal.pone.0353265.r003

**Authors:** Andrej Kielbassa, Andrej Kielbassa

22 Jun 2026

<p>Bond strength of debonded orthodontic brackets after different reconditioning and priming protocols: An in vitro study

PONE-D-25-59612R1

Dear Dr. Nguyen,

We’re pleased to inform you that your manuscript has been judged scientifically suitable for publication and will be formally accepted for publication once it meets all outstanding technical requirements.

Within one week, you’ll receive an e-mail detailing the required amendments. When these have been addressed, you’ll receive a formal acceptance letter and your manuscript will be scheduled for publication.

An invoice will be generated when your article is formally accepted. Please note, if your institution has a publishing partnership with PLOS and your article meets the relevant criteria, all or part of your publication costs will be covered. Please make sure your user information is up-to-date by logging into Editorial Manager at Editorial Manager® and clicking the ‘Update My Information' link at the top of the page. For questions related to billing, please contact billing support.

If your institution or institutions have a press office, please notify them about your upcoming paper to help maximize its impact. If they’ll be preparing press materials, please inform our press team as soon as possible -- no later than 48 hours after receiving the formal acceptance. Your manuscript will remain under strict press embargo until 2 pm Eastern Time on the date of publication. For more information, please contact onepress@plos.org.

Kind regards,

Andrej M Kielbassa

Academic Editor

PLOS One

Additional Editor Comments (optional):

Please note that I have double checked (R #4) your revised and re-submitted draft after both external Reviewers have recommended 'acceptance'.

Reviewers' comments:

Reviewer's Responses to Questions

**Comments to the Author**

1. If the authors have adequately addressed your comments raised in a previous round of review and you feel that this manuscript is now acceptable for publication, you may indicate that here to bypass the “Comments to the Author” section, enter your conflict of interest statement in the “Confidential to Editor” section, and submit your "Accept" recommendation.

Reviewer #1: All comments have been addressed

Reviewer #3: All comments have been addressed

Reviewer #4: All comments have been addressed

**********

2. Is the manuscript technically sound, and do the data support the conclusions?

The manuscript must describe a technically sound piece of scientific research with data that supports the conclusions. Experiments must have been conducted rigorously, with appropriate controls, replication, and sample sizes. The conclusions must be drawn appropriately based on the data presented.

Reviewer #1: Yes

Reviewer #3: Yes

Reviewer #4: Yes

**********

3. Has the statistical analysis been performed appropriately and rigorously?

Reviewer #1: Yes

Reviewer #3: Yes

Reviewer #4: Yes

**********

4. Have the authors made all data underlying the findings in their manuscript fully available?

The PLOS Data policy requires authors to make all data underlying the findings described in their manuscript fully available without restriction, with rare exception (please refer to the Data Availability Statement in the manuscript PDF file). The data should be provided as part of the manuscript or its supporting information, or deposited to a public repository. For example, in addition to summary statistics, the data points behind means, medians and variance measures should be available. If there are restrictions on publicly sharing data—e.g. participant privacy or use of data from a third party—those must be specified.

Reviewer #1: Yes

Reviewer #3: Yes

Reviewer #4: Yes

**********

5. Is the manuscript presented in an intelligible fashion and written in standard English?

PLOS ONE does not copyedit accepted manuscripts, so the language in submitted articles must be clear, correct, and unambiguous. Any typographical or grammatical errors should be corrected at revision, so please note any specific errors here.

Reviewer #1: Yes

Reviewer #3: Yes

Reviewer #4: Yes

**********

6. Review Comments to the Author

Please use the space provided to explain your answers to the questions above. You may also include additional comments for the author, including concerns about dual publication, research ethics, or publication ethics. (Please upload your review as an attachment if it exceeds 20,000 characters)

Reviewer #1: All Review comments are addressed. These were the comments from initial review:

Comment 1 – Suggest adding some definitions and importance of performance indicator parameters (i.e - SBS, ARI, bonding reliability etc), this could make the manuscript more coherent and user-friendly for broader audience.

Comment 2 – Discussion of Potential Enamel Risk with High SBS Groups

Location: Section 4, Page 20, Lines 337–340

While mentioned briefly in the Discussion, please expand on the clinical implications regarding enamel crack risk during debonding, and the balance between strong adhesion and safe removal procedures.

Comment 3 – Weibull Analysis Explanation

Location: Section 3.2 – Weibull Analysis, Page 12–13, Lines 224–235

The Weibull analysis is appropriate and informative. To improve clarity for readers, please add a brief explanation on how modulus m relates to clinical reliability

Comment 4 – Minor Formatting Improvements

A light editorial pass is recommended to ensure consistency in:

• Figure numbering and caption style (Figures 1–3)

• Table formatting

These edits will improve readability and professional presentation.

Reviewer #3: Thanks for addressing all my comments.

No further amendments are required.

The paper now looks suitable for publication.

Reviewer #4: - This revised and re-submitted draft would seem ready to proceed.

- Congratulations, and stay healthy!

**********

7. PLOS authors have the option to publish the peer review history of their article (what does this mean?). If published, this will include your full peer review and any attached files.

If you choose “no”, your identity will remain anonymous but your review may still be made public.

**Do you want your identity to be public for this peer review?**  For information about this choice, including consent withdrawal, please see our Privacy Policy.

Reviewer #1: **Yes:** Chinmay Potdar

Reviewer #3: No

Reviewer #4: No

**********

# Editor report: Acceptance letter

**DOI:** 10.1371/journal.pone.0353265.r004

**Authors:** Andrej Kielbassa, Andrej Kielbassa

PONE-D-25-59612R1

PLOS One

Dear Dr. Trang,

I'm pleased to inform you that your manuscript has been deemed suitable for publication in PLOS One. Congratulations! Your manuscript is now being handed over to our production team.

At this stage, our production department will prepare your paper for publication. This includes ensuring the following:

* All references, tables, and figures are properly cited

* All relevant supporting information is included in the manuscript submission,

* There are no issues that prevent the paper from being properly typeset

You will receive further instructions from the production team, including instructions on how to review your proof when it is ready. Please keep in mind that we are working through a large volume of accepted articles, so please give us a few days to review your paper and let you know the next and final steps.

Lastly, if your institution or institutions have a press office, please let them know about your upcoming paper now to help maximize its impact. If they'll be preparing press materials, please inform our press team within the next 48 hours. Your manuscript will remain under strict press embargo until 2 pm Eastern Time on the date of publication. For more information, please contact onepress@plos.org.

You will receive an invoice from PLOS for your publication fee after your manuscript has reached the completed accept phase. If you receive an email requesting payment before acceptance or for any other service, this may be a phishing scheme. Learn how to identify phishing emails and protect your accounts at https://explore.plos.org/phishing.

If we can help with anything else, please email us at customercare@plos.org.

Thank you for submitting your work to PLOS ONE and supporting open access.

Kind regards,

PLOS ONE Editorial Office Staff

on behalf of

Prof. Dr. med. dent. Dr. h. c. Andrej M Kielbassa

Academic Editor

PLOS One
